# Low-grade gliomas do not grow along white matter tracts: evidence from quantitative imaging

**DOI:** 10.1093/braincomms/fcaf157

**Published:** 2025-04-19

**Authors:** Philip Rauch, Matthias Gmeiner, Martin Aichholzer, Matthias Sterrer, Helga Wagner, Stefan Katletz, Carlo Serra, Petra Böhm, Michael Sonnberger, Nico Stroh, Stefan Aspalter, Kathrin Aufschnaiter-Hiessböck, Tobias Rossmann, Francisco Ruiz-Navarro, Maria Gollwitzer, Annette Leibetseder, Josef Pichler, Wolfgang Thomae, Raimund Kleiser, Andreas Gruber, Harald Stefanits

**Affiliations:** Department of Neurosurgery, Kepler University Hospital and Johannes Kepler University Linz, Linz 4040, Austria; Clinical Research Institute for Neuroscience, Faculty of Medicine, Johannes Kepler University Linz, Linz 4020, Austria; Department of Neurosurgery, Kepler University Hospital and Johannes Kepler University Linz, Linz 4040, Austria; Clinical Research Institute for Neuroscience, Faculty of Medicine, Johannes Kepler University Linz, Linz 4020, Austria; Department of Neurosurgery, Kepler University Hospital and Johannes Kepler University Linz, Linz 4040, Austria; Clinical Research Institute for Neuroscience, Faculty of Medicine, Johannes Kepler University Linz, Linz 4020, Austria; Department of Applied Statistics, Medical Statistics and Biometry, Johannes Kepler University, Linz 4040, Austria; Department of Applied Statistics, Medical Statistics and Biometry, Johannes Kepler University, Linz 4040, Austria; Department of Neurology, Kepler University Hospital and Johannes Kepler University, Linz 4020, Austria; Department of Neurosurgery, Clinical Neuroscience Center, University Hospital, University of Zurich, Zurich 8091, Switzerland; Machine Intelligence in Clinical Neuroscience (MICN) Lab, Department of Neurosurgery, Clinical Neuroscience Center, University Hospital Zurich, University of Zurich, Zurich 8091, Switzerland; Department of Neurosurgery, Kepler University Hospital and Johannes Kepler University Linz, Linz 4040, Austria; Institute of Neuroradiology, Kepler University Hospital and Johannes Kepler University, Linz 4020, Austria; Department of Neurosurgery, Kepler University Hospital and Johannes Kepler University Linz, Linz 4040, Austria; Clinical Research Institute for Neuroscience, Faculty of Medicine, Johannes Kepler University Linz, Linz 4020, Austria; Department of Neurosurgery, Kepler University Hospital and Johannes Kepler University Linz, Linz 4040, Austria; Clinical Research Institute for Neuroscience, Faculty of Medicine, Johannes Kepler University Linz, Linz 4020, Austria; Department of Neurosurgery, Kepler University Hospital and Johannes Kepler University Linz, Linz 4040, Austria; Clinical Research Institute for Neuroscience, Faculty of Medicine, Johannes Kepler University Linz, Linz 4020, Austria; Department of Neurosurgery, Kepler University Hospital and Johannes Kepler University Linz, Linz 4040, Austria; Clinical Research Institute for Neuroscience, Faculty of Medicine, Johannes Kepler University Linz, Linz 4020, Austria; Department of Neurosurgery, Kepler University Hospital and Johannes Kepler University Linz, Linz 4040, Austria; Clinical Research Institute for Neuroscience, Faculty of Medicine, Johannes Kepler University Linz, Linz 4020, Austria; Department of Neurosurgery, Kepler University Hospital and Johannes Kepler University Linz, Linz 4040, Austria; Clinical Research Institute for Neuroscience, Faculty of Medicine, Johannes Kepler University Linz, Linz 4020, Austria; Department of Neurology, Kepler University Hospital and Johannes Kepler University, Linz 4020, Austria; Institute of Internal Medicine and Neuro-oncology, Kepler University Hospital, Johannes Kepler University, Linz 4020, Austria; Institute of Internal Medicine and Neuro-oncology, Kepler University Hospital, Johannes Kepler University, Linz 4020, Austria; Department of Neurosurgery, Kepler University Hospital and Johannes Kepler University Linz, Linz 4040, Austria; Clinical Research Institute for Neuroscience, Faculty of Medicine, Johannes Kepler University Linz, Linz 4020, Austria; Institute of Neuroradiology, Kepler University Hospital and Johannes Kepler University, Linz 4020, Austria; Department of Neurosurgery, Kepler University Hospital and Johannes Kepler University Linz, Linz 4040, Austria; Clinical Research Institute for Neuroscience, Faculty of Medicine, Johannes Kepler University Linz, Linz 4020, Austria; Department of Neurosurgery, Kepler University Hospital and Johannes Kepler University Linz, Linz 4040, Austria; Clinical Research Institute for Neuroscience, Faculty of Medicine, Johannes Kepler University Linz, Linz 4020, Austria

**Keywords:** personalized medicine, glioma growth, vector deformation field, radial glia, white matter tract

## Abstract

Low-grade gliomas are infiltrative brain tumors that can lead to significant neurological deficits due to their invasive nature. The prevailing belief is that low-grade gliomas primarily disseminate along white matter tracts, but quantitative *in vivo* evidence supporting this concept is lacking. Clarifying their true growth patterns is essential for optimizing therapeutic strategies. We conducted a quantitative analysis of tumor growth patterns in a longitudinal cohort of 43 untreated patients with unigyral World Health Organization grade 2 or 3 gliomas, stratified by their anatomical locations within the neocortex, mesocortex and allocortex. Serial MRI scans were used to generate vector deformation fields, providing detailed three-dimensional representations of tumor evolution over time. These vector deformation fields were compared with diffusion tensor imaging data to assess the alignment of tumor growth with white matter pathways. Quantitative analysis revealed that low-grade gliomas do not predominantly expand along white matter tracts. Instead, they remain confined within specific anatomical boundaries, in respect to their topology of origin. Angular measurements and heat map analysis indicated that tumor growth is directed towards the subventricular zone and may follow their respective radial units. These consistent observations across different anatomical regions challenge the traditional model of glioma progression, suggesting that early-stage glioma expansion is closely governed by ontogenetic factors. In conclusion, this study provides the first quantitative evidence that phenotypical low-grade gliomas do not primarily follow white matter tracts but may instead be influenced by ontogenetic mechanisms. These insights necessitate a re-evaluation of existing models of glioma progression and underscore the importance of incorporating developmental aspects into treatment planning to enhance patient outcomes.

## Teaser

Rauch *et al*. provide the first quantitative evidence that Low Grade Gliomas do not primarily grow along white matter tracts. Instead, they follow an ontogenetically and anatomically driven growth pattern, challenging current models of tumor progression.

## Graphical Abstract

**Graphical Abstract:**
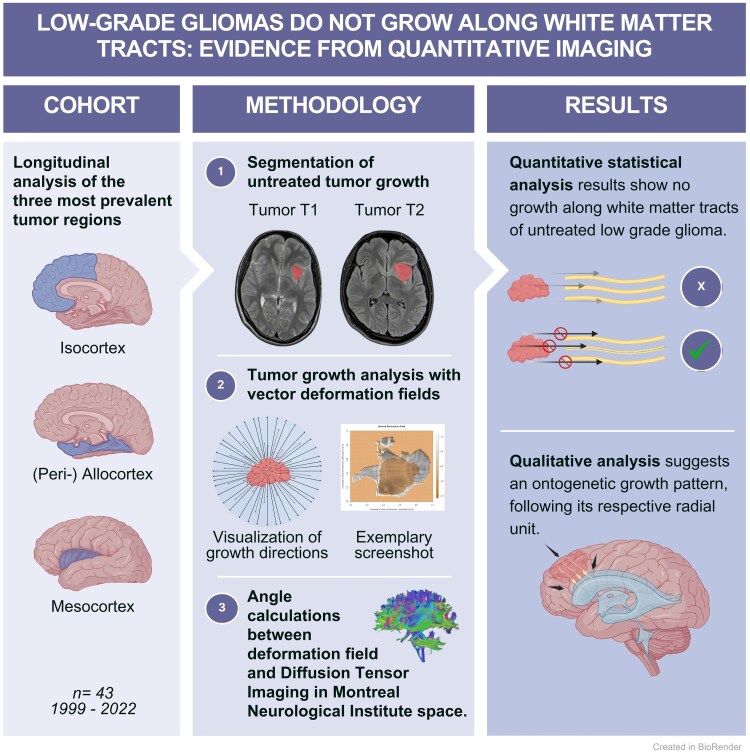

## Introduction

Understanding the growth patterns of low-grade gliomas (LGG) and intermediate-grade gliomas (for the purpose of simplification herein subsumed as LGGs) is a major yet unresolved challenge in neuro-oncology, with significant implications for patient management and treatment strategies. Traditionally, LGGs have been believed to extend predominantly along white matter tracts—a hypothesis that has endured despite the lack of robust *in vivo* evidence.^1,2^ While *in vitro* analyses suggest that white matter tracts could serve as conduits for LGG migration,^3-6^ direct macroscopic evidence remains limited, necessitating a re-evaluation of the mechanisms underlying LGG invasion and progression.

Emerging research points towards alternative models for glioma growth, highlighting factors beyond white matter infiltration. Recent findings in neuroepithelial tumors, using topographic probability mapping and clustering analyses, have demonstrated intrinsic radial organization consistent with Rakic *et al*.'s radial unit hypothesis^7-9^. This hypothesis posits that the cerebral cortex is organized into radial columns originating from proliferative zones during development. Akeret *et al*.^10-12^ provided evidence that gliomas might adhere to these developmental architectures, indicating that LGGs could follow ontogenetic patterns rather than strictly disseminating along white matter tracts. Additionally, interactions with neural precursor cells (NPCs) have been implicated in glioma progression. NPCs located in the subventricular zone (SVZ) secrete chemoattractant signals that may guide glioma cells toward these regions.^13,14^ This chemoattraction underscores the critical role of microenvironmental cues and developmental pathways in shaping LGG behaviour.

Direct histopathological validation of specific LGG growth models in humans is challenging due to ethical constraints on repeated sampling of unaffected brain tissue and the standard clinical focus on gross total resection or adjuvant therapy.^15,16^ These limitations highlight the necessity for advanced, non-invasive imaging techniques to elucidate the dynamic behaviour of LGGs.

Diffusion tensor imaging (DTI) has provided valuable insights into white matter structure and the impact of gliomas.^17^ However, its limitations—chiefly its static representation and vulnerability to confounding factors like mass effect and edema—necessitate complementary methods to understand dynamic growth.^18^

Vector deformation field (VDF) analysis represents a significant advancement, offering quantitative, three-dimensional mapping of tumor-induced changes over time. This approach directly measures growth dynamics, providing a detailed view of both direction and magnitude of tissue deformation.^19,20^ Combining DTI with VDF analysis yields a more comprehensive understanding of LGG growth, integrating spatial white matter changes with temporal growth trajectories. This integrated approach holds promise for improving patient outcomes by advancing both the clinical management and biological understanding of LGGs.

In this study, a diffeomorphic modelling technique was employed to generate VDFs from serial imaging of untreated (i.e. no prior surgical or adjuvant therapeutic interventions) LGGs at a high-volume center. Imaging data collected at initial diagnosis and prior to any interventions enabled the documentation of unaltered growth trajectories, resulting in what is, to our knowledge, the most comprehensive cohort analysis of the natural progression of low-grade gliomas.^21^ The LGGs were stratified based on their location in neocortical, mesocortical and allocortical regions to assess growth patterns across these distinct cerebral domains. Deformation fields derived from VDF analysis were systematically compared to DTI to challenge or confirm the traditional hypothesis of white matter tract-mediated progression, ultimately aiming to refine our understanding of LGG growth dynamics.

## Methods

**Figure 1:**
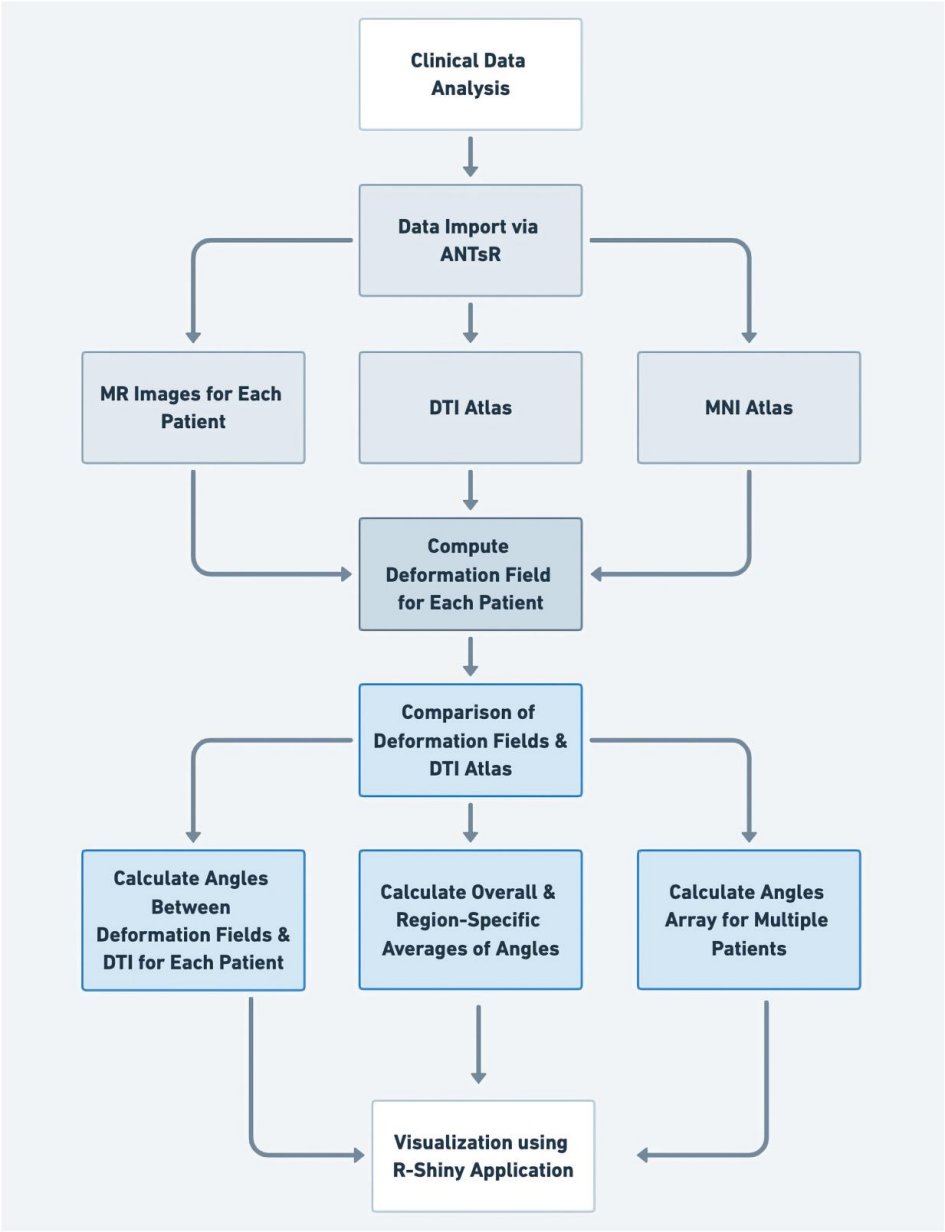
**Five-stage workflow.** This figure outlines a five-stage workflow for the analysis and representation of glioma progression. The process begins with the collection of clinical data, followed by the computation of deformation vectors. These vectors are then integrated with diffusion-tensor imaging (DTI) data and visualized on a standardized Montreal Neurological Institute (MNI) brain template to demonstrate the directional tendencies of tumor expansion. ANTsR, Advanced Normalization Tools.

The study was structured into five methodological stages, as illustrated in Fig. 1.

Ethics board approval was obtained prior to data acquisition from the local ethics committee (JKU-Ethikkommission, EK-2021-1042). All patients or their legal representatives gave their legal informed consent, and the study was conducted in accordance with the Declaration of Helsinki. The results are reported in accordance with the STROBE statement.

### Source population

We established a comprehensive retrospective database utilizing the CALUMMA system (RISC Software, Austria) to collect data from a sequential cohort of 355 patients diagnosed with WHO grade 2 or 3 gliomas at the Department of Neurosurgery, Kepler University Hospital and Johannes Kepler University, Linz, Austria from 1999 to 2022. From this cohort, 43 patients with histologically confirmed gliomas localized to the superior frontal gyrus, insular mesocortex or temporomesial allocortex, were identified, meeting the predefined inclusion and exclusion criteria. Clinicopathological details are presented in Supplementary Table 1.

Below is one way you could rephrase it for clarity and a more formal tone:

For each patient, two MRI protocols were required: Timepoint 1 (T1) was defined as the earliest MRI that fulfilled the prerequisite criteria (described below) at the time of diagnosis. Timepoint 2 (T2) was the final MRI obtained before the initiation of surgery or any adjuvant treatment.

Preoperative MRI scans underwent a qualitative assessment based on a modified topological and phylogenetic tumor extension protocol as proposed by Akeret *et al*.^12^ Anatomical structures considered merely displaced or edematous, without invasion, were categorized as unaffected. This was occasionally supplemented by post-surgical MRI findings. Volumetric segmentation of T2/FLAIR signal abnormalities was systematically performed on all suitable MRI scans at Kepler University Hospital. This process entailed a sequential evaluation by a senior neuroradiologist (M.S., > 30 years of experience) and neurosurgeons (P.R., H.S., M.A.), covering the entire timeline of MRI data for each subject. P.R., H.S. and M.A. performed the segmentations independently; in the event of discrepancies, M.S. served as mediator in accordance with previously published protocols.^22-24^. All segmentations received final approval from M.S. Pairwise Dice coefficients and Jaccard indices were calculated among the raters to objectively evaluate segmentation performance. This analysis was performed on a representative, randomly selected subset comprising 34% of the total cohort (see Supplemental Content C). We opted not to employ a deep learning–based segmentation method due to the heterogeneous availability of imaging sequences in the T1 scan sessions (e.g. potentially missing transversal T1, T1c, FLAIR or T2 sequences necessary for our deep learning algorithm), which would have reduced the eligible patient cohort. Instead, segmentations were performed using the semi-automated ImFusion Labels tool (ImFusion, 2022), supported by an interactive level-tracing utility for the initial delineation of T2/FLAIR anomalies.

### Inclusion criteria

- Histologically confirmed WHO grade 2 or 3 supratentorial glioma as per the WHO 2021 classification;
- preoperative MRI availability, encompassing T1 sequences with and without contrast, T2 and FLAIR;
- at least two successive FLAIR MRIs at a minimum interval of 2 weeks before surgery, without the commencement of adjuvant chemotherapy or radiotherapy;
- an absence of neoadjuvant treatments or prior cranial surgeries;
- a unigyral glioma confined to the superior frontal gyrus, mesocortical insula or temporomesial allocortical regions.

### Exclusion criteria

- Distortion or suboptimal image quality in MRI sequences that could potentially compromise segmentation analysis, such as susceptibility artefacts or patient movement;
- pre-existing structural cerebral anomalies that might impede precise segmentation, such as hypoxic scars or other pathological conditions like additional tumors or arteriovenous malformations;
- incomplete clinical data, particularly pertaining to treatment history or histopathological specifics;
- presentation of multifocal glioma or tumor spread beyond the predetermined anatomical confines at the time of diagnosis;
- high-grade features like ring-shaped contrast enhancement or central necrosis at radiological diagnosis or increased perfusion signal at diagnosis.

### Vector field calculation

The progressive dynamics of gliomas were analysed by comparing sequential T2- or FLAIR-MRI-based VDF calculations with a DTI atlas or patient-specific DTI, against the backdrop of the MNI standardized brain template.^25^ Preprocessing involved conforming patient-specific imaging data to a consistent volumetric space with dimensions of 181 × 217 × 181 voxels. The methodology and comprehensive workflow are shown in Supplemental Content A. The study used four primary neuroimaging datasets:

1. Temporal MR patient images: Sequential MR images capture the evolution of gliomas, which provided the basis for the deformation field calculations.
2. DTI Atlas: The DTI atlas provides a voxel-resolution map of water diffusion. Furthermore, it ensures high angular resolution without subject dependent heterogeneity.
3. MNI Atlas: Use of the MNI template ensures anatomical consistency across patient-specific imaging data.
4. Patient-specific DTI: Comparison of DTI and Vector Deformation Fields for additional subject-specific analysis.

Tumor segmentation masks were overlaid onto the MNI standard brain template to visualize glioma spatial distribution at specific timepoints.

For the preprocessing step, SPM12 (version 7771) was used to generate the transformation fields from a patient’s T1 3D MRI to MNI space. As described by Kurth *et al*., the tissue probability map (TPM) was symmetrized by averaging the original TPM with its left-right mirrored version. These transformation fields were subsequently applied to the aligned segmentation images,^26^ and the transformed volumes were resampled to a voxel resolution of 1 mm^3^ using SPM12. In cases where a tumor was located in the right hemisphere, the normalized segmentation was mirrored left-right to ensure all tumors were positioned in the left hemisphere. This approach generated tumor mask segmentations in MNI space, aligning with the ICBM 2009c Nonlinear Symmetric atlas.^27^

This process involved the generation of a color map that aggregated voxel-wise frequencies of gliomas across the patient cohort. For each voxel, a binary variable indicating presence of glioma was created to enable a detailed and quantifiable analysis of the glioma distribution patterns. The DTI component contained data from the IIT Human Brain Atlas (v5.0) (Supplemental Content A). A noted spatial disparity between the DTI dataset and the MR images necessitated a harmonization process, ensuring dataset compatibility and integrative analysis fidelity.

### Harmonization of neuroimaging data for voxel-wise analysis

In voxel-wise neuroimaging analyses, congruence in voxel dimensions, count and image orientation is paramount. For this study, image orientation consistency was ensured across all images using the sform function from the R-package oro.nifti. Voxel details were verified within the NIfTI file headers. However, disparities in the overall dimensionality existed as follows:

- MR images: 181 × 217 × 181 voxels
- DTI atlas: 182 × 218 × 182 voxels
- MNI image: 182 × 218 × 182 voxels

The additional voxels present in the DTI atlas and the MNI image, relative to the MR images, required truncation to facilitate accurate cross-modality comparisons. The key challenge was determining which segments of the data array to remove without compromising the spatial correlation.

To address this issue, images had to be transformed to ensure the same orientation in world-space coordinates across all images. The extraneous voxels in the DTI and MNI datasets (i = 181, j = 217, k = 181) mapped to coordinates beyond the MR image bounds (x = −91, y = 91, z = 109) and were thus excluded from analyses. The workflow for patient-specific DTI is shown in Supplemental Content B.

### Quantification of tumor progression through deformation field analysis

**Figure 2:**
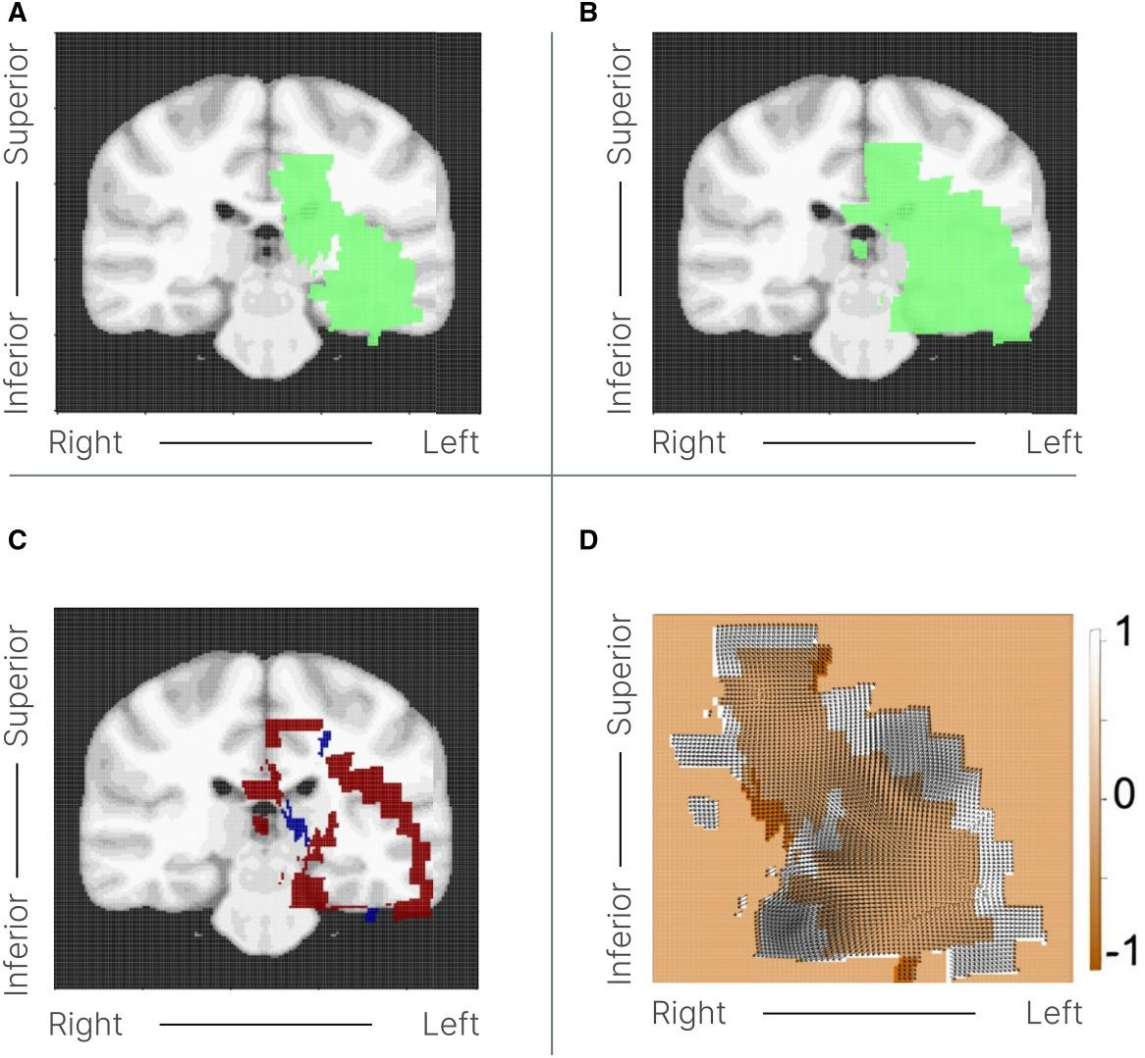
**Spatiotemporal mapping of glioma dynamics in patient X on the MNI brain template.** This figure demonstrates the spatial and temporal changes in glioma volume for Patient X highlighting an allocortical growth pattern. (**A** and **B**) Show the segmented tumor regions at the initial (T1) and follow-up (T2) timepoints, respectively, overlaid on a coronal slice of the MNI brain template. (**C**) Presents the differential map (T21), highlighting regions of tumor growth (in red) with a value of 1, indicating ‘positive growth.’ Areas marked in blue with a value of −1 represent ‘negative growth,’ attributed to minor segmentation variability rather than actual tumor regression. (**D**) Offers a two-dimensional representation of deformation vectors on a voxel-wise basis, capturing the direction and magnitude of tumor-related changes between T1 and T2. The arrows at each voxel illustrate the deformation direction, with the central orange mask indicating the initial tumor boundary (T1) and the white outline depicting changes observed at the follow-up (T21), providing a detailed assessment of the tumor's dynamic behaviour over time. Similar to **C** the heat map denotes regions with a value of +1 as ‘positive growth,’ reflecting actual tumor expansion, whereas values of −1 indicate ‘negative growth’.

Tumor growth was analyzed based on the transformation from the tumor at time point T1 to time point T2. To accurately capture the typically anisotropic growth of irregularly shaped tumors, vector deformation fields were computed. The values in each voxel of a vector deformation field represented the displacement in all three dimensions required to match the MRI at T1 with the MRI at T2 (Fig. 2).

### Comparative analysis of glioma growth

The primary objective was to investigate whether there was alignment between the vectors indicating glioma growth and the diffusion directions denoted in the DTI atlas. The glioma growth for each patient was represented as a multidimensional array with the dimensions of 181 × 217 × 181 × 3, indicating the direction of tissue deformation for each voxel. In a similar fashion, the DTI atlas is a multidimensional array with identical dimensions, representing the first eigenvectors. Given that each voxel corresponds to a three-dimensional vector within the same anatomical reference frame, it was feasible to calculate the angular alignment for each voxel.

Three-dimensional arrays of angular alignment values were computed for each voxel across all patients. The alignment between tumor growth vectors and the principal eigenvectors derived from the DTI atlas was calculated for each voxel, expressed as an angle ranging from 0 to 90 degrees. This angle served as a measure of how closely the direction of tumor growth followed or deviated from the white matter tracts. To evaluate directional preference, we tested the hypothesis that angular alignment below 20 degrees indicates preferential growth along the white matter tracts. Detailed descriptions of the calculations and angle transformations are provided in Supplemental Content C.

Subsequently, these angular values were averaged across patients, taking into account the tumor location (e.g. Allocortex, Mesocortex, Isocortex). Thus, for a given voxel, only the angular measurements associated with glioma presence were averaged, ensuring specificity to tumor growth.

To allow for interactive inspection to enable the interactive inspection of the derived vector fields and angular alignments across various patient cohorts and brain regions, the R-shiny App ‘Tumor Growth Analysis’ was developed. A screenshot of the application interface, along with an explanation of its functionality, is provided in Supplementary Fig. 1.

We further compared Absolute Growth, Growth per Day and Growth Factors across the three brain regions. Three key measures were computed for each patient:

- Absolute Growth: This measure represents the change in glioma volume between T1 and T2 calculated as the volume at Time Point 2 (T2) minus the volume at Time Point 1 (T1).
- Growth per Day: This rate was determined by dividing the Absolute Growth by the number of days between the two timepoints.
- Growth Factor: The ratio of glioma volume at Time Point 2 (T2) to Time Point 1 (T1). The percentage increase of the tumor volume is given as: (Growth Factor−1) × 100.

### Statistical analysis

To assess whether tumor growth aligns with white matter tracts, we compared the observed distribution of the acute angles between the growth vectors and the principal eigenvectors derived from DTI with the distribution expected under isotropic growth, i.e. equal growth in all directions. Angles were computed for all subjects across all voxels to evaluate the alignment with white matter tracts (Supplementary Fig. 7). For an intuitive graphical assessment, histograms of these angles were overlaid with the theoretical distribution under the isotropic growth model. Subsequently, to test whether the proportion of angles <20 degrees differed from the expected proportion under isotropic conditions, we employed a Binomial test. Detailed mathematical derivations and analyses are provided in Supplemental Content C.

## Results

### MRI analysis results

#### Overall glioma growth

Pairwise segmentation performance was quantified using Dice coefficients and Jaccard indices among three independent raters (A–C) (see Supplementary Fig. 8). Mean Dice coefficients were 0.897 for A–B, 0.896 for A–C and 0.907 for B–C (SD: 0.031, 0.031 and 0.027, respectively), while the mean Jaccard indices were 0.814, 0.813 and 0.831 (SD: 0.051, 0.051 and 0.045, respectively).

In this study, patient-specific glioma measurements were obtained at two time points (T1 and T2). Preliminary analysis indicated that absolute tumor growth is less variable in the Allocortex region compared to other regions. Median Growth per Day is similar in all regions, with the smallest variability in the Mesocortex region. Notably, there was a divergence between patients exhibiting the highest Growth per Day and those with the largest Absolute Growth, highlighting the heterogeneity of tumor progression rates among individuals (Supplementary Fig. 6B).

To assess differences in growth metrics among brain regions, we performed a Kruskal–Wallis H-test due to the non-normal distribution of the data and the requirement for multi-group comparison. The test yielded H = 1.54 and *P* = 0.46, indicating no statistically significant difference in ‘Growth per Day’ between the allocortex, mesocortex and isocortex (*P* > 0.05).

Similarly, for the comparison of ‘Growth Factor’ across regions, the Kruskal–Wallis H-test yielded H = 2.62 and *P* = 0.27, demonstrating no statistically significant difference among the allocortex, mesocortex and isocortex (*P* > 0.05).

### Time intervals

The distribution of time intervals between measurement points T1 and T2 across the Allocortex, Isocortex and Mesocortex regions was calculated. The mean intervals were 1030.0 days ± 1643.3; (23–4453) for the Allocortex, 380.7 days ± 796.8; (14–3013) for the Isocortex and 515.0 days ± 1088.4; (20–3640) for the Mesocortex.

Normality tests (Shapiro–Wilk) showed non-normal distributions for all regions (*P* < 0.001). Given these results, a Mann–Whitney U-test was employed, which identified a statistically significant difference between the Isocortex and Allocortex (*P* = 0.044), while comparisons between other regions were not significant (*P* > 0.05).

### Visualization and analysis of deformation fields

In the current study, deformation fields served as an analytical tool to analyse glioma growth. It is essential to note that the results were consistent regarding the directions and lengths of growth vectors across all patients. Angular alignments for each patient were then visualized using the R Shiny application. The distributions of these angles are shown as histograms to facilitate easier interpretation. Additionally, for more intuitive understanding, angles are displayed on an MNI template on a voxel-by-voxel basis, using varying color hues to represent different angles (Supplementary Fig. 1).

### Statistical analysis of angular distributions

**Figure 3:**
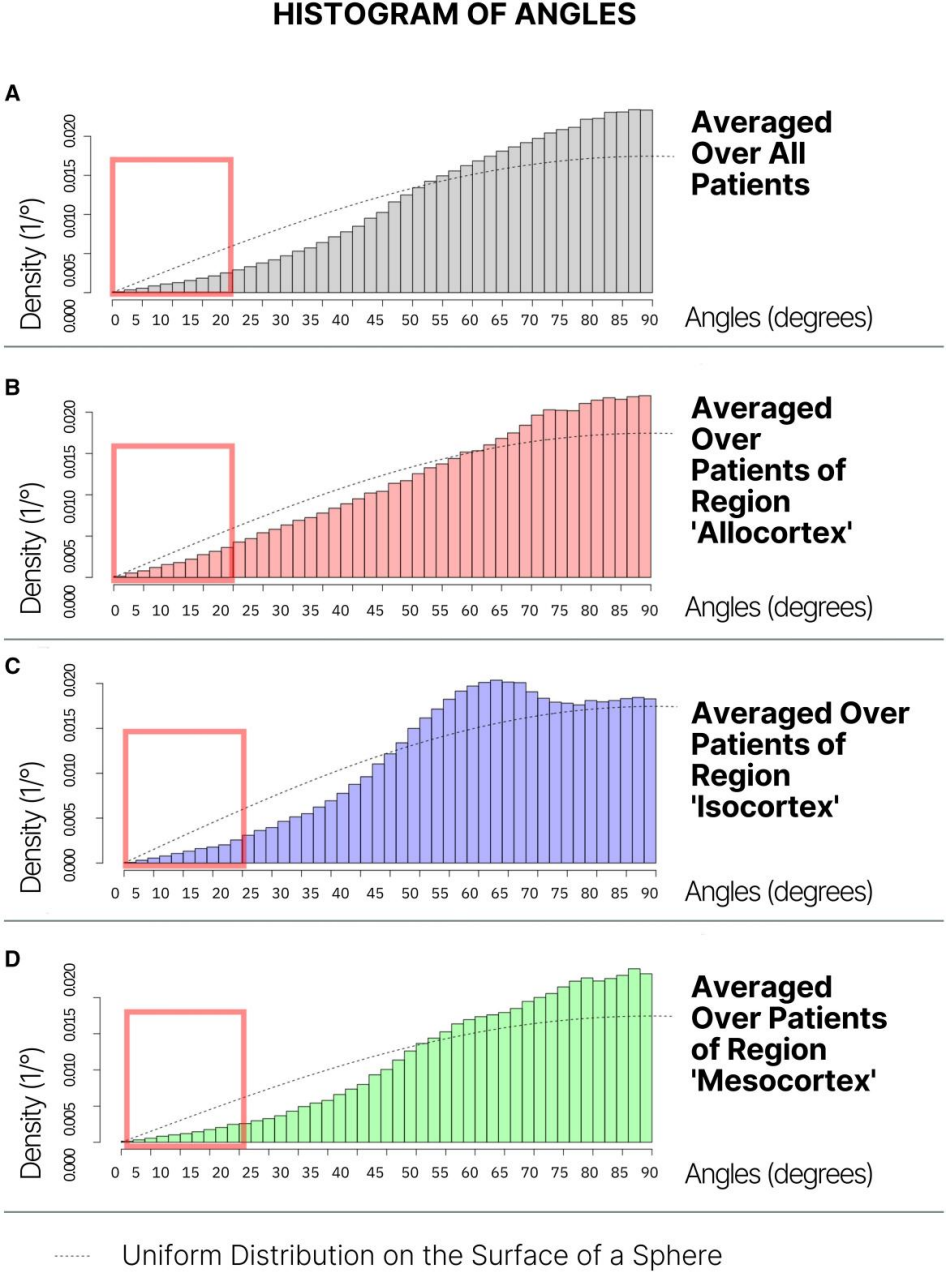
**Voxel-Wise analysis of tumor growth directions in low-grade gliomas.** This figure shows average angle distributions (θ⋆) for the entire patient cohort (**A**) and region-specific transformed angles (**B**-**D**). The *x*-axis represents the measured angles (in degrees), while the *y*-axis shows the normalized probability density, with units of 1/°. This normalization ensures that the area under the histogram sums to 1, providing a relative frequency distribution of the angles. The red square marks the 0–20% region, wherein angular aggregation would be indicative of alignment with white matter tracts. The dotted line shows the distribution of the angles under isotropic tumor growth. (**A**) Distinctly shows that the theoretical frequency of isotropic growth under 20 degrees substantially exceeds the observed frequency. This discrepancy contradicts the theory of growth along white matter tracts, where angles in the range of 0–20 degrees would have a significantly higher frequency. Herein, region-specific histograms (**B**-**D**) for individual tumor areas also highlight that the distribution has a mode at 90 degrees and θ⋆ a similar left-skewed trend.

In our voxel-wise analysis of tumor growth directions, we visualized the average angle distributions (θ and θ⋆) across our entire patient cohort using histograms for intuitive analysis (Fig. 3). We defined angles <20 degrees as representing alignment between the calculated tumor growth directions and the white matter tracts. Accordingly, we computed the proportions of angles smaller than 20 degrees for both the collective dataset and individual regions, comparing these to the expected angle distribution under isotropic growth using a binomial test. Additionally, calculations were carried out for each patient individually.

The observed proportion *h* was derived by dividing the number of angles <20 degrees across all patients by the total number of angles, yielding a value of 0.0352. Visually, the histograms suggest that the probability of an angle below 20 degrees is considerably lower in our patient cohort than the theoretical probability under assumed isotropic growth. This observation is consistent with the calculated values, where the expected proportion under isotropic growth (p_0_ = 0.0603) is significantly larger than the observed relative frequency (*h* = 0.0352 h).

These results show that early-stage LGGs do not primarily propagate along white matter tracts. Instead, we observed a left-skewed distribution, which can be interpreted as a tendency for perpendicular growth with respect to white matter tracts, irrespective of tumor topology. Detailed calculation methods are provided in Supplemental Content C.

The use of patient-specific DTI data yielded similar results, validating our decision to employ a DTI atlas for enlarging our cohort size and consistent high angular resolution (Supplementary Fig. 5). Additionally, it is important to note that angular alignments calculated exclusively for T21 deformation fields showed no significant difference from those derived from the complete tumor vector field, underlining the consistency of our findings.

### Macroscopic growth patterns

A heat map distribution across all patients and regions was further investigated to quantify macroscopic growth patterns. To navigate the multidimensional nature of MRI data, color maps were used to represent glioma occurrence frequencies voxel-wise and to provide insights into glioma locations at different time points, stratified according to their respective ontogenetic sites (Supplementary Figs 2–4). MRI data superimposed on the MNI template highlighted distinct patterns in different cerebral regions.

### Isocortex

**Figure 4:**
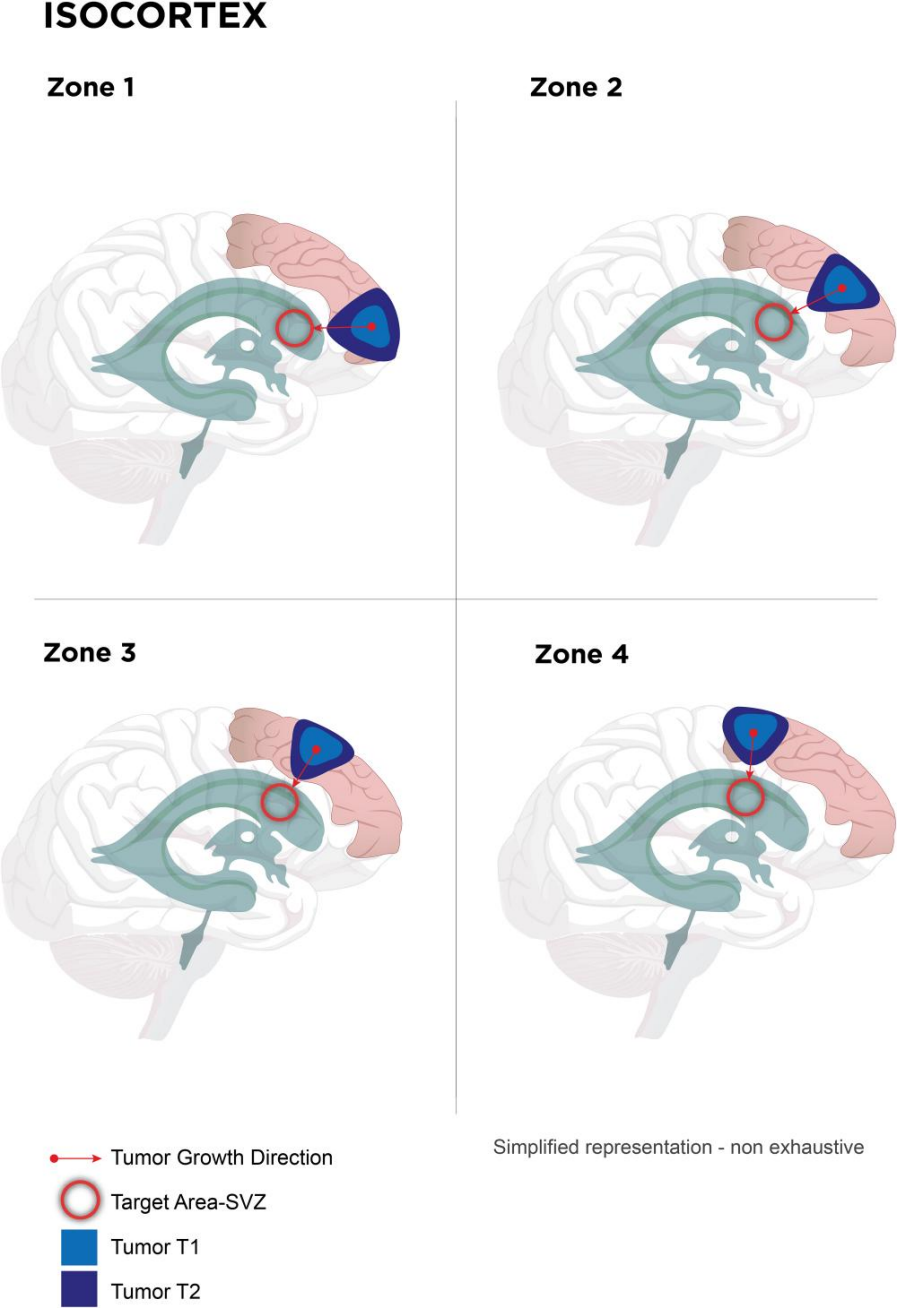
**Regional tumor growth patterns within the isocortex in relation to the subventricular zone (SVZ).** Schematic representation of tumor distribution across four regions (Zone 1–4) of the isocortex. Tumor states at two time points (T1 and T2) are illustrated in red to yellow gradients, with lighter shades representing greater tumor volumes. Red circles indicate regions in proximity to the SVZ that potentially attract tumor growth. Arrows depict the direction of tumor expansion towards these SVZ-adjacent regions. The schematic highlights the spatial relationship between tumor development and its directional preference towards SVZ-associated regions.

**Figure 5:**
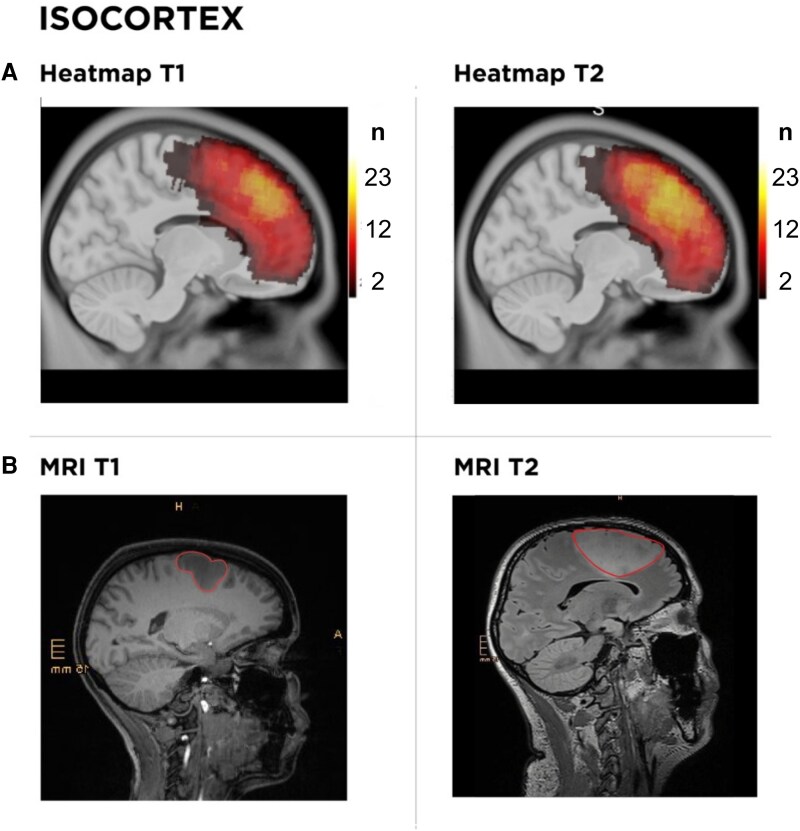
**Spatiotemporal analysis of tumor density and structural progression within the isocortex.** (**A**) Heatmaps depicting tumor density distributions at two time points (T1 and T2) within the isocortex. The color scale ranges from red to yellow, indicating low to high tumor density, respectively. The heatmaps show the progression of the tumor over time, with increasing densities shifting towards the ventricular surface area. The heat map scale indicates the number of patients (N) corresponding to each value. (**B**) MRI scans demonstrating the longitudinal evolution of a Zone 3 tumor at time points T1 and T2. Tumor boundaries are highlighted in red, delineating changes in size and location within the isocortex over time. The MRI images offer a complementary view to the heatmaps, confirming spatial expansion and volumetric increase, showcasing a radial glial growth pattern.

Isocortical tumors, represented by the superior frontal gyrus, displayed a predilection for growth toward the ventricular system or subventricular zone, with negligible invasion into adjacent gyri like the middle frontal and cingulate. Four distinct growth zones could be differentiated, potentially representing specific radial units (Zone 1: *n* = 3, Zone 2: *n* = 8, Zone 3: *n* = 9, Zone 4: *n* = 3; Figs 4 and 5, Supplementary Fig. 2).

### Mesocortex

**Figure 6:**
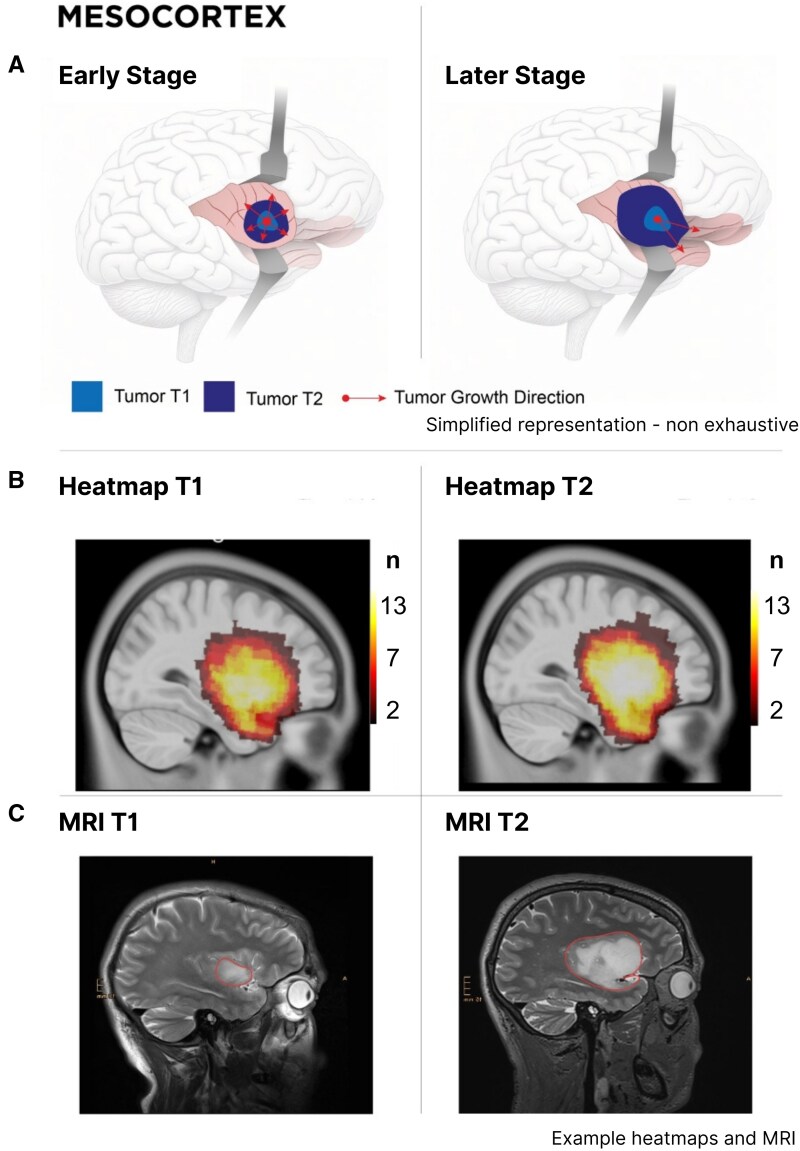
**Tumor progression within the mesocortex from early to later stages.** (**A**) Schematic representation of glioma development in the mesocortex. During early stages, tumors are predominantly localized within the insula (upper left). In later stages, larger tumors (upper right) expand into adjacent mesocortical regions, including the orbitofrontal cortex and temporal Poles. Arrows indicate the direction of tumor growth. This schematic emphasizes the transition from localized insular growth to broader mesocortical involvement, rather than growth along white matter tracts. (**B**) Heatmaps of tumor density at two time points (T1 and T2) within the mesocortex. The color gradient from red to yellow indicates increasing tumor density via number of patients (N) corresponding to each value. At T1, tumors are concentrated within the insula. By T2, the heatmaps reveal denser and broader tumor infiltration extending into the orbitofrontal cortex and temporal Poles. (**C**) MRI scans showing the structural characteristics of the tumor at T1 and T2. The red outlines delineate the tumor boundaries, illustrating the stereotypical pattern of mesocortical tumor growth over time.

Mesocortical tumors (Fig. 6, Supplementary Fig. 3) showed marked superior–inferior and anterior–posterior growth, respecting the boundaries of the ventral striatum. These growths remained confined within the mesocortex, sparing median structures and limited infiltration to adjacent structures only to the transitional orbital and temporal pole regions.

### (peri-)allocortex

**Figure 7:**
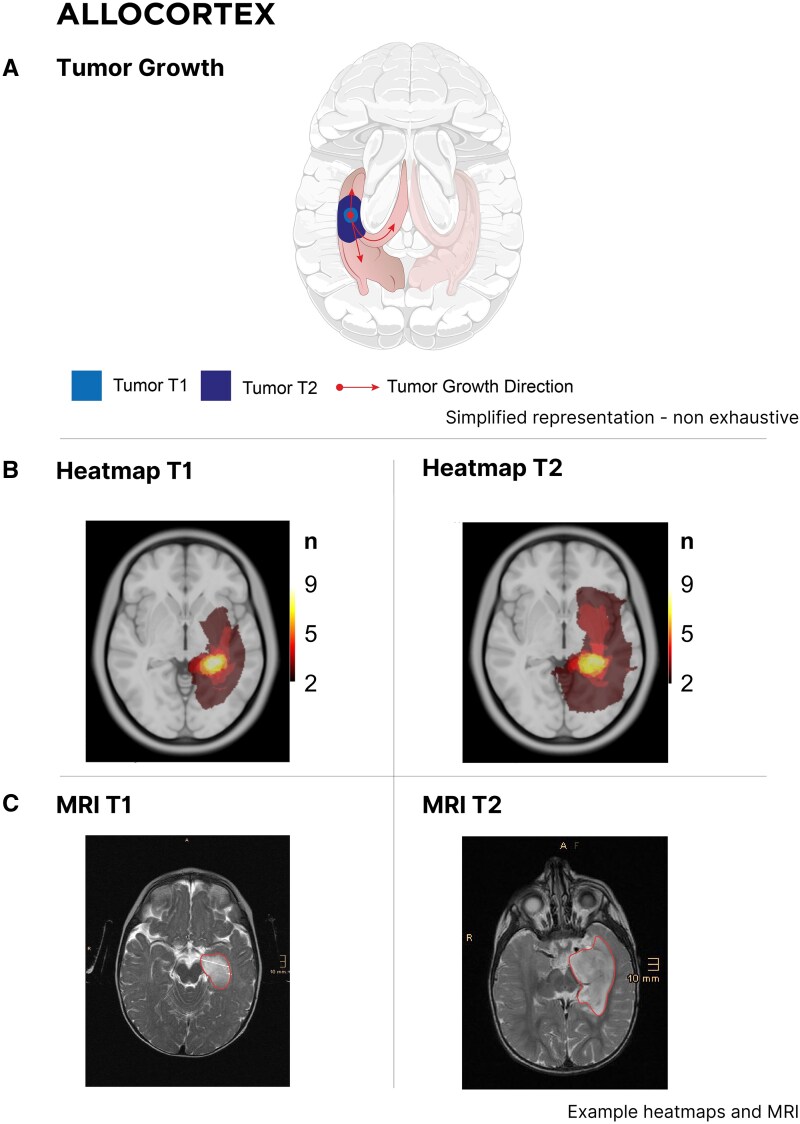
**Tumor progression within the allocortex.** (**A**) Schematic representation of glioma progression confined to the (peri-)allocortex. At the initial stage (T1), tumors originate within allocortical regions. In later stages (T2), the growth remains restricted to the allocortex, without infiltrating adjacent neocortical areas. Red arrows indicate the stereotypical direction of growth, illustrating its confinement within allocortical structures. (**B**) Heatmaps illustrating tumor density at two time points (T1 and T2) within the allocortex. The color scale from red to yellow denotes increasing density via the number of patients (N) corresponding to each value. The T1 heatmap shows initial tumor localization, while the T2 heatmap reveals increased density and expansion confined to the allocortex, with no involvement of neighboring neocortical regions. (**C**) MRI scans depicting tumor boundaries (red outlines) at T1 and T2. The images confirm volumetric growth confined to the (peri-)allocortex without evidence of spread into adjacent neocortical areas.

Tumors within the (peri-)allocortex (Fig. 7, Supplementary Fig. 4) exhibited similar restrained infiltration patterns, sparing the neocortical areas and affecting only the anterior transition zone of the temporal pole (Brodmann Area 38) beside the parahippocampal formation and fornix.

## Discussion

In this study, we identify and characterize distinct growth patterns of low-grade gliomas, demonstrating consistent stereotypy and adherence to specific topological constraints throughout their natural progression. Using dynamic vector deformation and heat map analysis, our investigation of 43 untreated, longitudinally evaluated, unigyral LGGs challenges the conventional view that early-stage LGG growth predominantly follows white matter tracts. By focusing on the three most prevalent anatomical regions, we aimed to understand the varying influence of human ontogeny and local white matter anatomy on glioma morphology.^5,10^ Herein, we quantify the *in vivo* morphological changes of a rare cohort of LGGs across successive imaging intervals, offering novel insights into the natural history of these tumors. Importantly, direct histopathological validation of specific LGG growth models in humans is impeded by ethical restrictions on repeated sampling of unaffected brain tissue and by clinical priorities that emphasize achieving gross total resection or administering adjuvant therapy immediately after diagnosis. Consequently, this longitudinal imaging approach serves as a critical alternative, enabling a deeper understanding of LGG progression while circumventing the limitations associated with invasive validation methods. Additionally, this method facilitated a comprehensive macroscopic examination of tumor expansion patterns, allowing systematic comparison with white matter structures via DTI.^19,20,28,29^ To promote reproducibility and further glioma research, we provide a detailed procedural protocol.

Recent advancements in microscopic studies and *in vitro* analyses have robustly demonstrated the interaction and growth of glioma cells along white matter fibre tracts. The current array of *in vitro* models, including nerve fibre cultures with electrophysiologically active neurons, nanomaterials, brain slice cultures, organoids and microfluidic chips, has significantly improved the precision of brain tumor research.^4^ For example, Wang *et al*. observed a preferential colonization of white matter tracts by glioma stem cells, marked by CD133 + Notch1 + expression, in regions expressing Jagged1 at glioma invasion margins.^6^ Despite the detailed understanding of glioma cell migration along white matter and perivascular spaces at the microscopic and *in vitro* level, caution must nevertheless be exercised when extrapolating these findings to the macroscopic level of human gliomas, particularly lower-grade variants. Such caution is warranted due to the fundamental disparities between murine and *in vitro* models, the predominant use of glioblastoma cell lines and the complex milieu of human *in vivo* conditions. To the best of the author’s knowledge, no quantitative evaluation of longitudinal LGG growth in humans has been conducted thus far.

The butterfly glioma, characterized by bilateral corpus callosum infiltration, is frequently used to illustrate macroscopic white matter infiltration in human gliomas.^1,30^ However, this specific anatomical pattern is not observed in low-grade gliomas and only represents a small percentage (6.2%) of all glioblastomas.^11^ Additionally, to the authors’ awareness, there have been no documented instances of glioma progression patterns, irrespective of WHO grade, extending from the frontal to occipital regions in alignment with the inferior frontooccipital fascicle. Similarly, there have been no reported cases of glioma development encircling the insula along the trajectory of the superior longitudinal fascicle, extending from the frontal operculum to the middle temporal gyrus. Moreover, it warrants attention that, as reported by Serra *et al*.,^31^ midbrain gliomas generally spare the corticospinal tract. This trait not only renders these gliomas more amenable to surgical resection but also suggests that glioma growth properties do not inherently favour invasion into white matter tracts.

To address this inconsistency, Akeret *et al*.^12^ expanded upon the findings of Yasargil *et al*.^34^ and the principle of topographical selective vulnerability (pathoclisis)—a concept first introduced by H.J. Scherer in the 1930s and 1940s^32,33^—to propose an ontogenetic model for glioma expansion. This model, derived from unsupervised clustering of neuroepithelial tumors, suggests that tumor growth patterns are linked to cellular differentiation levels and may depend on their respective radial units. Specifically, lower-grade, well-differentiated neuroepithelial tumors exhibited static growth, localized displacement and expansion along the radial axis. In contrast, only after undergoing malignant transformation to a mesenchymal phenotype, these tumors predominantly exhibited migration along white matter tracts or perivascular invasion.

Furthermore, Qin *et al*. demonstrated that pleiotrophin, in complex with SPARC, SPARCL1, and HSP90B, is secreted by neural precursor cells—predominantly located in the subventricular zone—thereby enhancing glioma cell invasion toward this region. Recent studies on neural-glioma synaptic connections suggest that gliomas not only interact with, but also exploit neural networks to facilitate their growth. By adhering to ontogenetic boundaries, lower-grade gliomas may utilize developmental pathways, including radial glial migration and neural stem cell niches, as mechanisms for their expansion.

**Figure 8:**
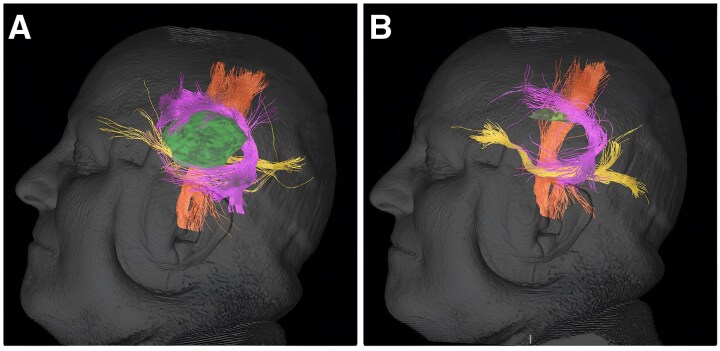
**Resection of an insular low-grade glioma with preservation of white matter tracts.** (**A**) Depicts an insular low-grade glioma (green) adjacent to key white matter tracts, including the arcuate fasciculus (magenta), inferior fronto-occipital fasciculus (yellow) and corticospinal tract (orange). The tumor is confined to the mesocortex without evidence of infiltration into these tracts, suggesting that low-grade gliomas do not preferentially grow along white matter pathways. (**B**) Shows the postoperative outcome following a 95% resection performed with awake craniotomy. Postoperative imaging reveals the preservation of white matter tracts, with neuropsychological assessments demonstrating no deficits. Minor changes in tract appearance are attributed to postoperative edema. These findings indicate that low-grade gliomas predominatnly respect white matter boundaries, supporting the feasibility of safe resection in eloquent brain regions.

While the propensity of low-grade gliomas to disrupt white matter integrity is clinically acknowledged and potentially detrimental to the functional connectome,^35-37^ this observation alone does not suffice as conclusive evidence of an intrinsic growth predilection for these pathways. It is important to recognize that white matter integrity can in most cases be preserved even during extensive tumor resections, especially when facilitated by direct electrostimulation^38-40^ (Fig. 8). By repetitive activation or disinhibition of white matter tracts with an electric probe during surgery, surgeons can prevent new neurological deficits despite (supra-)total or near-total tumor removal.

In this patient series, we demonstrate consistent patterns of glioma growth that adhere to anatomical boundaries, guided by established histopathological frameworks such as those of Brodmann and Talairach, which may be consistent with the radial unit hypothesis (Figs 4–7). Herein, advanced DTI and VDF analysis revealed no preferential growth along white matter tracts. Contrarily, angular analysis revealed pronounced growth at approximately 90 degrees relative to the DTI, indicating that the tumors tended to grow perpendicularly rather than along the white matter tracts, regardless of the phylogenetic site analyzed (Figure 3). This observation aligns with the fact that, in preoperative imaging, white matter tracts are often distorted but not yet fully infiltrated at the borders of low-grade glioma (Fig. 8).^41^ Mesiotemporal gliomas, herein referred to as allocortical, were confined to the (peri-)allocortical domain and expanded preferentially along the hippocampal formation, eschewing invasion into adjacent isocortical regions (Fig. 7). Similarly, tumors located in the superior frontal gyrus—representative of neocortical LGGs in this study—showed growth patterns confined to the gyrus, while sparing the underlying mesocortical cingulum and advancing toward the ventricles, indicative of a ventriculopetal growth trajectory. We could discern four prominent growth foci which may be indicative of a separation of the superior frontal gyrus into distinct radial units (Figs. 4 and 5). Insular gliomas, classified as mesocortical (Fig. 6), demonstrated expansive and diffuse growth within the mesocortical regions, extending from superior to inferior directions and anteriorly into the transitional cortices of the orbitofrontal cortex via the orbital pole and into the transitional cortex of the temporal pole (Brodmann 38). For comparability, we focused exclusively on gliomas arising solely in the insula, thereby excluding opercular extensions. Consequently, longitudinal opercular infiltration was not observed in our cohort; however, this does not preclude its occurrence, as opercular involvement has been described previously.^34^ While it is plausible that tumor progression follows superficial u-fibres between the opercula and the insula in these cases, the precise mechanism—whether reflecting histocompartment boundaries or white-matter pathways—remains debated.

Recent microsurgical and anatomical investigations, including those by Demirtaş *et al*.^42^ underscore how the medial opercula remain insufficiently characterized from both histological and functional standpoints. This incomplete understanding complicates the determination of whether opercular extension arises from infiltration along u-fibres or via transitional cortical zones. In our experience, opercular extension, when present, typically remains confined to the medial (insular) aspect of the opercula, with full lateral opercular invasion usually seen only in more advanced disease. Given the rarity of such presentations and the limited availability of long-term, untreated follow-up data, we elected not to include them in this study. Furthermore, due to uncertainties regarding the precise histological composition of these regions, interpreting these tumors beyond recognizing a distinct pattern would be speculative. Future research should investigate and compare involvement of frontal subregions—particularly the orbital, opercular and triangular parts of the inferior frontal gyrus—in a multicentre setting, ensuring an adequately large patient population for robust analysis.

Further, while the uncinate fascicle is often involved in anterior insular gliomas, we argue that this is attributable to its thin structure and superficial proximity to transitional cortices rather than to a preferential, white matter–centric growth pattern. The dorsal extension of tumor growth into the insular cortices—with stereotypical sparing of major tracts such as the IFOF and arcuate fascicle—supports a model of tumor expansion governed by radial glia–guided developmental trajectories and intrinsic histocompartmentalization rather than by selective propagation along individual white matter tracts.

In this study, the application of vector deformation analysis to low-grade glioma research revealed distinct patterns when compared to those reported by Esmaeili *et al*.^19^ in their glioblastoma study. Notably, while Esmaeili *et al*. observed a predominant rightward skew of deformation vector angles (within the 0–20-degree range relative to diffusion tensors) in glioblastoma, indicative of growth patterns aligned with white matter tracts, our analysis of low-grade gliomas presented a predominant leftward skew (within the 90-degree range). This divergence could likely be attributed to the inherent differences in the methodologies adopted by the two studies. Primarily, their focus on glioblastoma, characterized by a higher degree of cellular dedifferentiation compared to low-grade glioma, likely influence their respective growth patterns. Secondly, they utilized T1-weighted imaging with contrast, opposed to our use of Fluid-Attenuated Inversion Recovery (FLAIR) imaging. FLAIR imaging is generally more effective in representing glioma infiltration, although it does introduce challenges, particularly in high-grade gliomas, such as difficulty in distinguishing actual tumor boundaries from peritumoral edema—a challenge less prevalent in low-grade gliomas. Additionally, analysis of Esmaili *et al*. did not account for the initial anatomical site of diagnosis and included tumors from the corpus callosum, a site not typically involved in low-grade gliomas, thereby limiting the applicability of their findings to this study context.

Recognizing the inherent limitations in comprehensively addressing all possible growth patterns and tumor localizations, we conducted a detailed examination of three of the most prevalent tumor locations in LGG.^43^ By considering ontogenetic factors and assessing the potential variability in white matter's anatomical susceptibility to glioma growth,^5,44^ the study thus aimed to achieve a cohort variability that is representative for an accurate depiction of LGG growth morphology. Therefore, this study deliberately excluded larger multigyral tumors to maintain comparability, as these tumors likely represent a more advanced disease stage or a higher level of cellular dedifferentiation.^11^

The size of the presented cohort is a noteworthy limitation, primarily due to the rarity and the now historic ‘wait and see’ approach. These factors constrain the recruitment of larger sample sizes, which may have restricted our ability to detect differences in growth behaviour among LGG subgroups. Future research should aim to integrate molecular biological signatures with ontogenetic growth patterns to further elucidate the factors driving LGG progression. Despite these limitations, to our knowledge, this study constitutes the largest cohort to date for evaluating growth patterns in anatomically specific, treatment-naïve lower-grade gliomas.^45^

A methodological discussion point is the use of a DTI atlas compared with patient-specific DTI data. To evaluate the impact of this choice on our results, we conducted additional analyses using a subset of patient-specific DTI datasets (Supplementary Fig. 5). However, no significant differences were observed compared to those using the DTI atlas. It is noteworthy that using separate warping algorithms (DTI-TK for DTI and SPM12 for T1w) for this purpose can potentially induce minor inter-modality misalignments that could affect voxel-wise angle quantification. Consequently, the authors opted for the DTI atlas to include a larger patient base and to ensure consistent high angular resolution across patients, aiming for a more comprehensive analysis and accurate results. The robustness and reproducibility of the results from the deformation analysis suggest that this technique is a significant advancement in analyzing glioma growth behaviour. Also, single-tensor DTI is unable to fully resolve crossing or ‘kissing’ fibres, which may introduce localized biases in principal eigenvector estimates. Nevertheless, as our analysis focuses on macro-scale tumor deformations across multiple anatomical regions, such localized confounders are unlikely to obscure a robust tract-aligned growth pattern. Future investigations should consider advanced diffusion imaging techniques, such as diffusion spectrum imaging (DSI) or probabilistic DTI, to further explore tumor–tract interactions, particularly in regions with complex fibre geometry. Despite expert segmentation, minor registration inaccuracies can still occur even with complex nonlinear transformations, potentially leading to small errors in deformation maps. To ensure precision, each scan was critically reviewed by multiple specialists post-registration.

## Conclusion

Our study introduces a novel quantitative approach that challenges the conventional view of low-grade glioma growth along white matter tracts. This paradigm shift has significant clinical implications. Recognizing that LGGs expand within defined anatomical constraints allows for more precise surgical interventions, optimizing tumor resection while potentially anticipating future glioma behaviour. In radiotherapy and chemotherapy, treatments can be tailored to the tumor's context-specific growth patterns, enhancing efficacy by targeting regions at higher risk of infiltration.

The use of VDF analysis provides a powerful tool for characterizing glioma growth, serving as a robust alternative to conventional DTI atlases in predictive modeling. This approach has the potential to refine patient stratification in clinical trials, tailoring therapeutic interventions to the unique growth dynamics of each tumor.

## Supplementary Material

fcaf157_Supplementary_Data

## Data Availability

The statistical code utilized for creating the deformation field models, R Shiny application, and angular alignment visualizations presented in this manuscript is accessible at https://github.com/PRauch1/Vector-Field.git. The code for the segmentation software, being a part of ImFusion's proprietary framework, is not publicly available. Due to privacy rights related to patient data, MRIs of patients cannot be shared openly, but they may be provided upon reasonable request. For further inquiries or specific requests, the corresponding author is available to respond as needed.
