# Supplementary material for: Low-grade gliomas do not grow along white matter tracts: evidence from quantitative imaging
Source: Brain Commun. 2025 Apr 19;7(3):fcaf157. doi: 10.1093/braincomms/fcaf157 (PMC12053163; doi:10.1093/braincomms/fcaf157)
Supplement: fcaf157_Supplementary_Data [file fcaf157_supplementary_data.docx]

**Supplemental Content A**

**Data Import**

The neuroimaging data used in this study are formatted as Neuroimaging Informatics Technology Initiative (NIfTI) files. For the import of these files, the R software packages ANTsR (version 0.5.7.5) and oro.nifti (version 0.11.4) were used. ANTsR acts as a wrapper, leveraging the Rcpp package to facilitate access to the Advanced Normalization Tools (ANTs) and the Insight Segmentation and Registration Toolkit (ITK) C++ core within the R environment. This package provides comprehensive capabilities including image segmentation and registration, as well as imaging-specific adaptations of principal component analysis and canonical correlation analysis. Detailed instructions for the installation of ANTsR are provided in Appendix A.

In addition, the oro.nifti package was used for the input and output of medical imaging data. It offers compatibility with various formats beyond NIfTI, such as Analyze. This facilitated a broad applicability in the processing and analysis of neuroimaging data.

**Download and Installation of ANTsR**

Since the ANTsR package is currently only hosted on Github and not on the more common CRAN platform, it requires a different method of installation. Additionally, this installation process requires specific operating systems to work properly. There are several ways to install it, and the method that has been successful for Windows 11 users is detailed here:

1. Download and install ’Ubuntu22.04.2LTS’ from <https://ubuntu.com/download/desktop>

2. Open the corresponding terminal and install ’R’
3. Check the version of R: R Version 4.1
4. Download the needed binary files (for the corresponding R version) from Github

- ANTsR: ANTsR 0.5.7.4 R x86 64-pc-linux-gnu R4.1.tar.gz from https://github.com/ANTsX/ANTsR/releases
- ANTsRCore: ANTsRCore 0.7.4.9 R x86 64-pc-linux-gnu R4.1.tar from https://github.com/ANTsX/ANTsRCore/releases
- ITKR: ITKR 0.5.3.3.0 R x86 64-pc-linux-gnu R4.1.tar.gz from https://github.com/stnava/ITKR/releases

5. Install the binary files via the R command:
install .packages(repos=”<Path of Binary File>/<Name of Binary File>”)

6. Load the package via the R command: library (ANTsR)

**Workflow**

Given that ANTsR is not compatible with Windows, the operating system of the used computer, it becomes necessary to execute certain segments of the code on an alternative platform. Specifically, the tasks related to data importation and the calculation of the deformation field must be conducted elsewhere.

The outlined sequence below delineates the steps required to import the data, generate the deformation fields, calculate the angular alignments, and visualize the outcomes. This process is divided into two parts: the initial segment is conducted on an Ubuntu virtual machine, while the subsequent segment is carried out on a Windows operating system. Each subtask is detailed with the respective function and file names, accompanied by the actual R code used in each step.

**Image Registration Utilizing ANTsR**

In the initial phase of analysis, pairs of MR images for each patient were loaded into R using the antsImageRead() function and stored as antsImage class objects. For a given patient, images from the first and second time points were designated as T1 and T2, respectively. The voxel-wise difference between T1 and T2 was computed, generating a new image, T21, where:

*T*21=*T*2−*T*1

Here, T21 represented a deformation image with the original dimensions, where each voxel was assigned one of three values: 1 (indicating positive tumor growth), 0 (indicating no change), or -1 (indicating tumor regression).

The antsRegistration() function facilitates the image registration, acting as a wrapper to transpose the arguments for processing by the underlying C++ implementation. It utilizes 'fixed' (T1) and 'moving' (T2) images to determine the mapping essential for deformation field calculation. The registration process was guided by the 'Symmetric Normalization' transformation model, with mutual information serving as the similarity metric. The parameters within antsRegistration() were maintained at default settings.

The output from antsRegistration() includes a list containing 'fwdtransforms', which details the transformation vectors defining the deformation field. As ANTsR operates within the LPS (Left-Posterior-Superior) coordinate system, a correction was necessary to align the deformation vectors with the LAS (Left-Anterior-Superior) orientation of the DTI and MNI images. This was achieved by inverting the second component of each vector, thus realigning positive values to indicate anterior growth and negative values to indicate posterior growth.

Top of Form

Bottom of Form

Part 1: Ubuntu Virtual Machine

1. Import the data as NIfTI files via ANTsR on Ubuntu

• For each patient 2 MR images
• DTI atlas
• MNI data of the standard human brain

1. Compute the deformation field for each patient via ANTsR on Ubuntu — Ubuntu get file name(), Ubuntu perform registration()
2. Save the results as .rda files on Ubuntu — Ubuntu export results.R

B Workflow 53 Part 2: Local Operating System (Windows):

1. Copy the results to the the local folders
2. Import the results to the local machine using R
   — import_DTI atlas.R, import_MNI.R, import_results(), import_multiple results.R, — check_axis orientation(), get_metadata.R
3. Compare the deformation field and the DTI atlas
   1. (a)  Calculate the angles between the deformation fields and the DTI for each patient.

— calculate angle between vectors(), calculate angles array for patient()

- 1. (b)  Calculate the overall average and the region-specific averages of the angles

— calculate angles array for multiple patients()

- 1. (c)  Save the results as rda files

— save angles arrays.R

1. Visualize the results using an R-Shiny Application
   1. (a)  Load the data (DTI, MR Images, deformation fields, MNI, computed angles)
   2. (b)  Define functions for proper visualization

— slice image 3d to 2d(), visualize results()

- 1. (c)  Show the results in the App — app.R

**DTI Atlas**

The DTI component used contained data from the IIT Human Brain Atlas (v5.0), herein the 'Mean DTI template' released on 2019-05-10 at 20:42, comprising:

1. IITmeanTensor.nii: This dataset contains the complete tensor values of the DTI template, arranged in a matrix with dimensions of 182 x 218 x 182 x 6, each representing the six tensor matrix values unique to each voxel.

2. IITmeanL1.nii: Herein, the principal eigenvalues are provided for each voxel within the mean DTI template, offering insights into the diffusion intensity.

3. IITmeanV1.nii: Access is given to the principal eigenvectors of the mean DTI template, with a 3D vector descriptor for each voxel.

4. IITmeanFA.nii: This file renders Fractional Anisotropy (FA) maps along with the principal eigenvector orientations, where positive vector component values correspond to the anatomical directions of Left, Anterior, and Superior (LAS), and negative values to their opposites.

**Subject-specific DTI-transformation to MNI**

1. Brain Extraction

Input: NIfTI file

Command: `bet data dti_data_brain -m`

2. Manual Correction of Brain Mask

Tool: MRICroGL

Process: Manual inspection and correction of the brain mask generated in step 1.

3. Eddy Current and Motion Correction

Inputs: Original NIfTI file, Corrected Brain Mask (dti_data_brain_mask_corrected)

Command: ```bash eddy --imain=data --mask=dti_data_brain_mask_corrected --acqp=acqparams.txt --index=index.txt --bvecs=bvec --bvals=bval --out=dti_data_eddy ```

4. Tensor Fitting

Inputs: Eddy-corrected image (dti_data_eddy), Corrected Brain Mask (dti_data_brain_mask_corrected), Eddy-rotated vectors (eddy_rotated_bvecs)

Command: ```bashdtifit -k dti_data_eddy -o dti -m dti_data_brain_mask_corrected -r eddy_rotated_bvecs -b bval```

5. Conversion to DTI-TK Compatible Volumes

Inputs: FSL-generated DTI eigensystem volumes

Command: `fsl_to_dtitk dti`

Verification: `TVglyphView -in dti_dtitk`

6. Rigid Alignment to DTI Template

Inputs: MNI atlas (template), DTI volume (dtitk)

Command: ```bash dti_rigid_reg template dti_dtitk EDS 4 4 4 0.01```

7. Affine Alignment

Inputs: MNI atlas (template), Rigidly Aligned DTI Volume (dti_rig)

Command: ```bash dti_affine_reg template dti_rig EDS 4 4 4 0.01```

8. Deformable Alignment

Inputs: MNI atlas (template), Rigidly & Affinely Aligned DTI Volume (dti_aff)

Command: ```bash dti_diffeomorphic_reg template dti_aff template_mask 1 6 0.002 ```

Resampling (if necessary):

Command: ```bash TVResample -in dti_diffeo -size 182 218 182 ```

Note: Flip the image if necessary to ensure tumors are on one side.

9. Calculation of Eigenvectors

Tool: MATLAB's "Tools for NIfTI and ANALYZE image"

10. Follow the steps in (Part 2. (6)), No additional steps (i.e., tumor masking) were necessary during visual inspection.

**Alternative approach for calculating patient-specific DTIs:**
Instead of performing angle calculations in MNI space, it is possible to perform VDF calculations directly in patient space. Depending on the specific research hypothesis, this approach may be more time efficient, and it eliminates the need to warp the DTI data to MNI space. However, for population comparisons, this approach could lead to compromised results.

**Supplemental Content B**

**Interactive Visualization through an R Shiny Application**


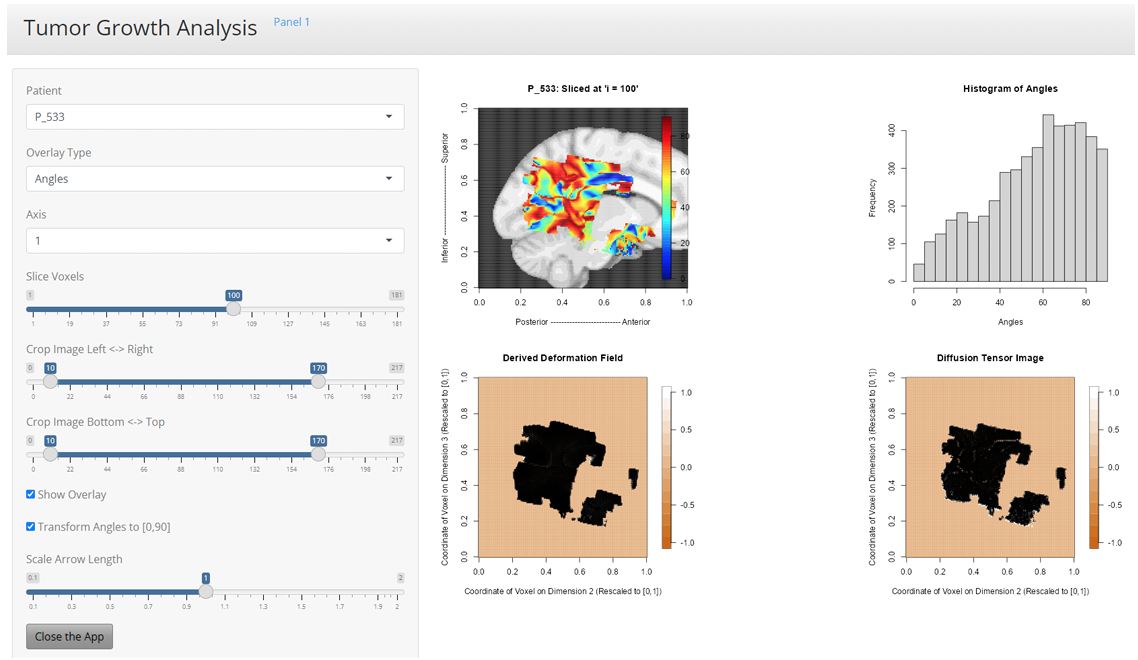


**Supplementary Figure 1: *Exemplary Screenshot:* Interface and Functionality of the Neuroimaging Analysis Tool.** This figure illustrates the application's sidebar, enabling users to select from patients or grouped regions like 'Allocortex', ‘Mesocortex’ and 'Isocortex'. Overlay options such as 'Angles' and 'Time Points Delta' are accessible via dropdown menus, along with choices for the analysis axis. Interactive scroll-bars allow for precise slice selection and image cropping. Checkboxes enable toggling of overlay displays and selection between angles θ and transformed angles θ⋆. The right panel of the interface displays four plots: the top-left shows the standard brain with overlays, the top-right highlights overlay distributions, and the lower plots depict vector fields, including the deformation field and DTI atlas, facilitating comprehensive brain structure and function. In these visualizations, a blue hue represents near-perfect alignment, with angles close to 0 degrees, while a red hue indicates vectors that are nearly perpendicular, with angles nearing 90 degrees. Accompanying each heatmap, histograms are provided to show the distribution of angles within the respective image slices.

**
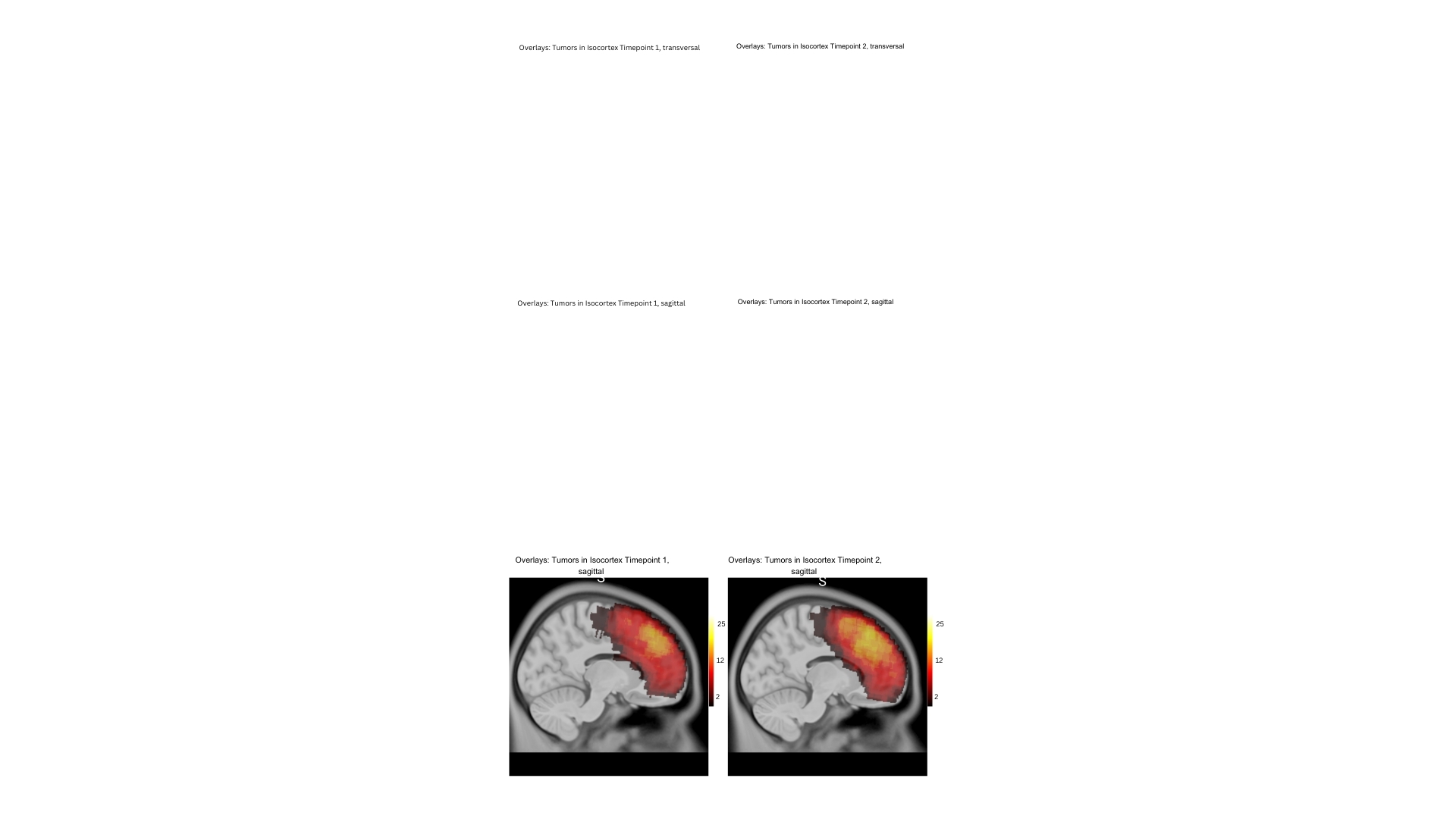

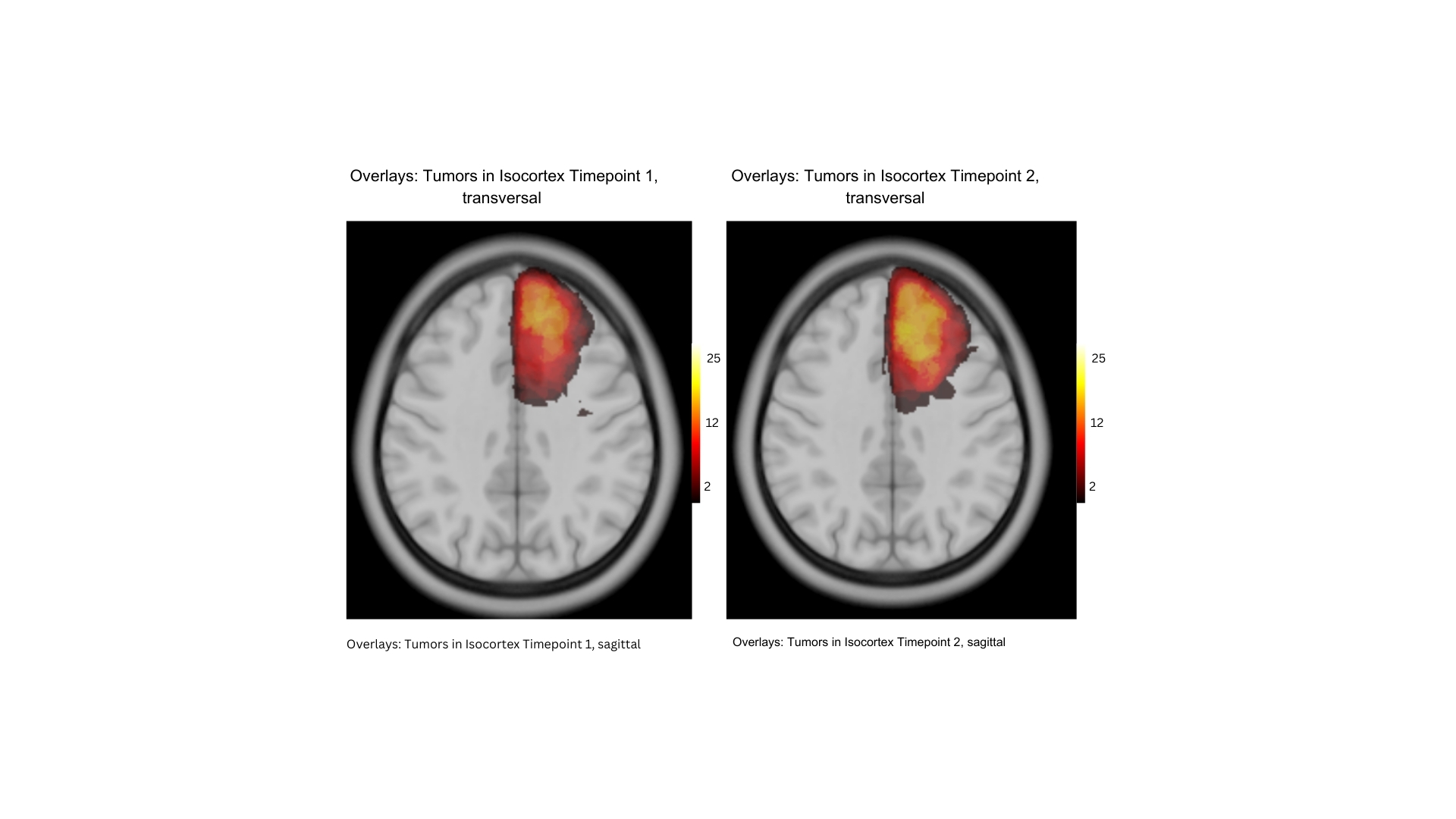
Heat Map Analysis of Macroscopic Growth Patterns in LGG**

n

n

n

n

**
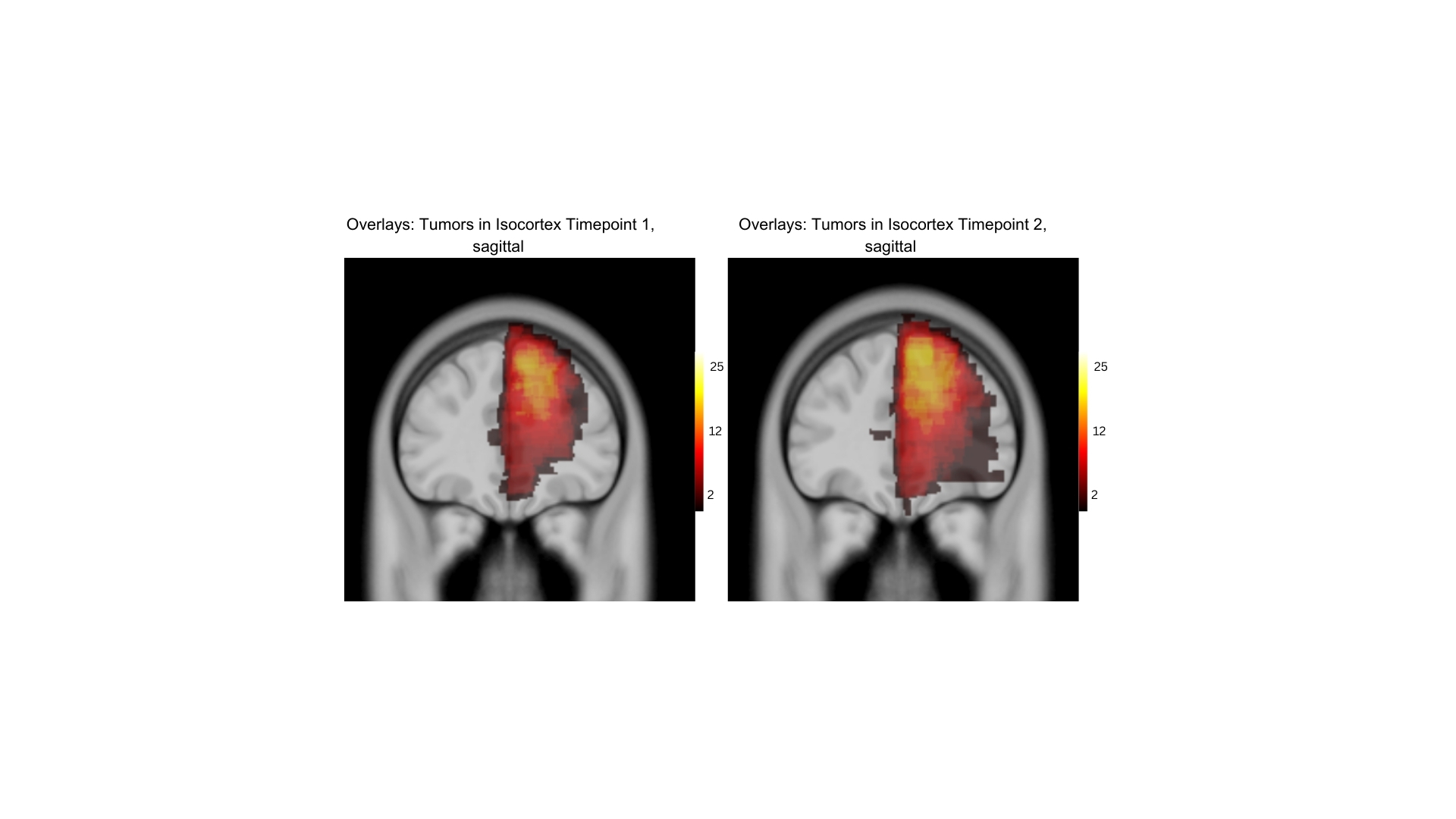
**

n

n

**Supplementary Figure 2: Neocortical Glioma Distribution and Dissection in the Superior Frontal Gyrus**

Transversal, sagittal and coronal MRI views, along with corresponding dissection images, demonstrate the frequency distribution of neocortical gliomas in the superior frontal gyrus. The color maps on standard MNI brain images quantify voxel-wise tumor occurrences, with a growth trend towards the ventricles. The legend decides the color-coded glioma frequencies (the number of patients (n) corresponding to each value), with Timepoints 1 and 2 indicating progression.


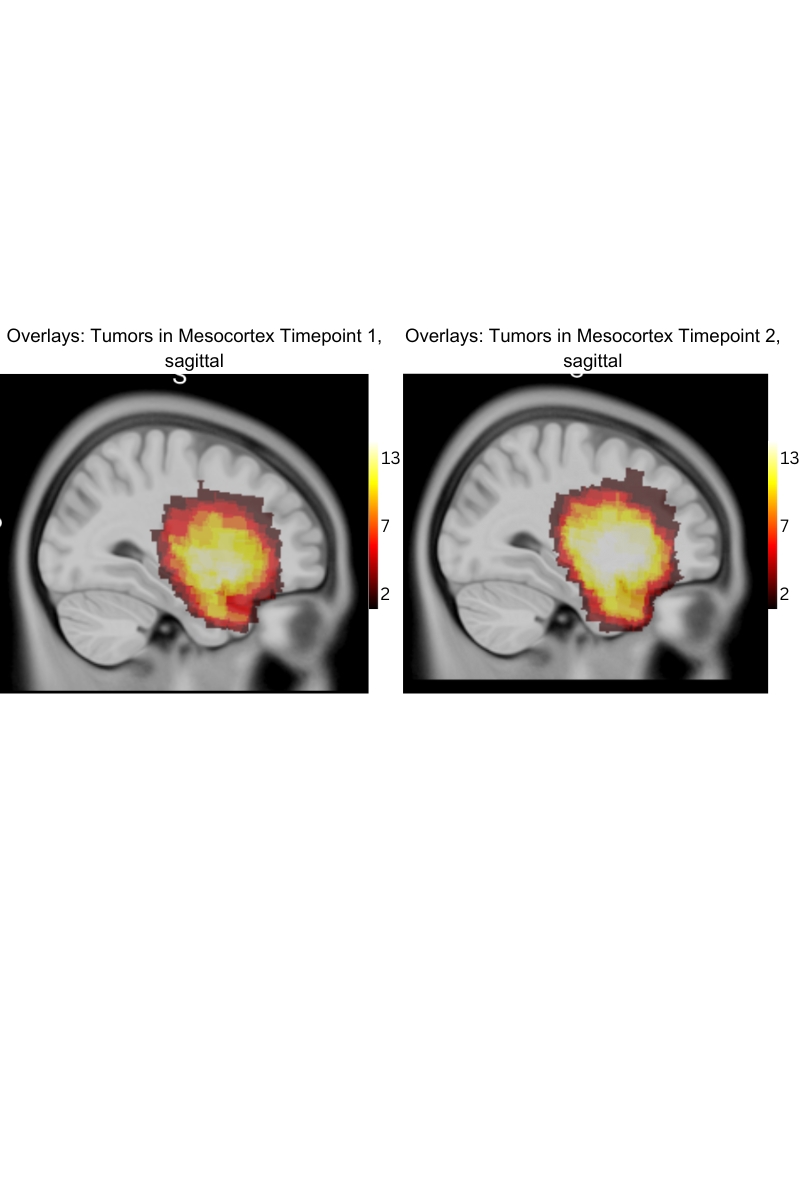

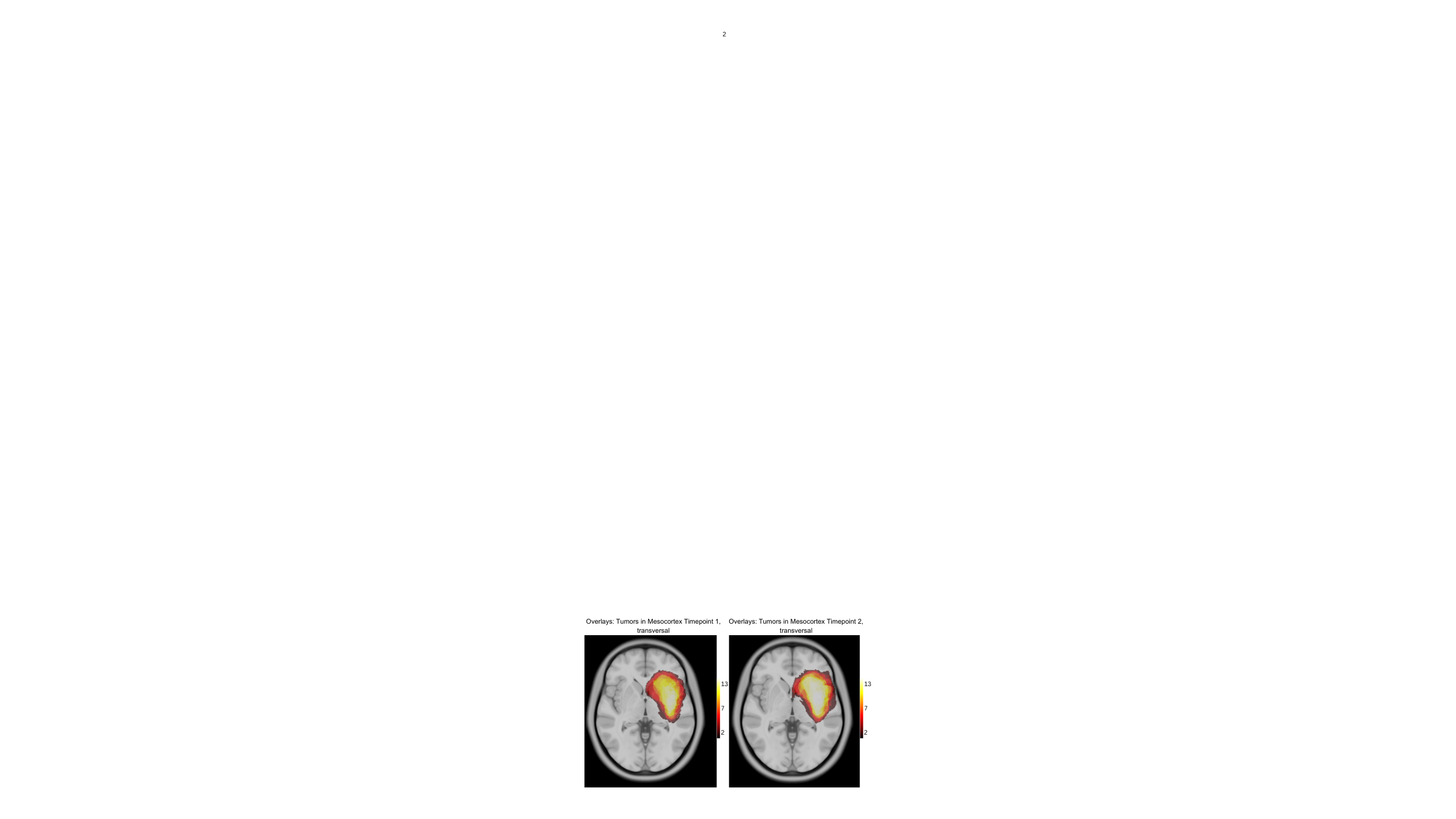


n

n

n

n

**Supplementary**
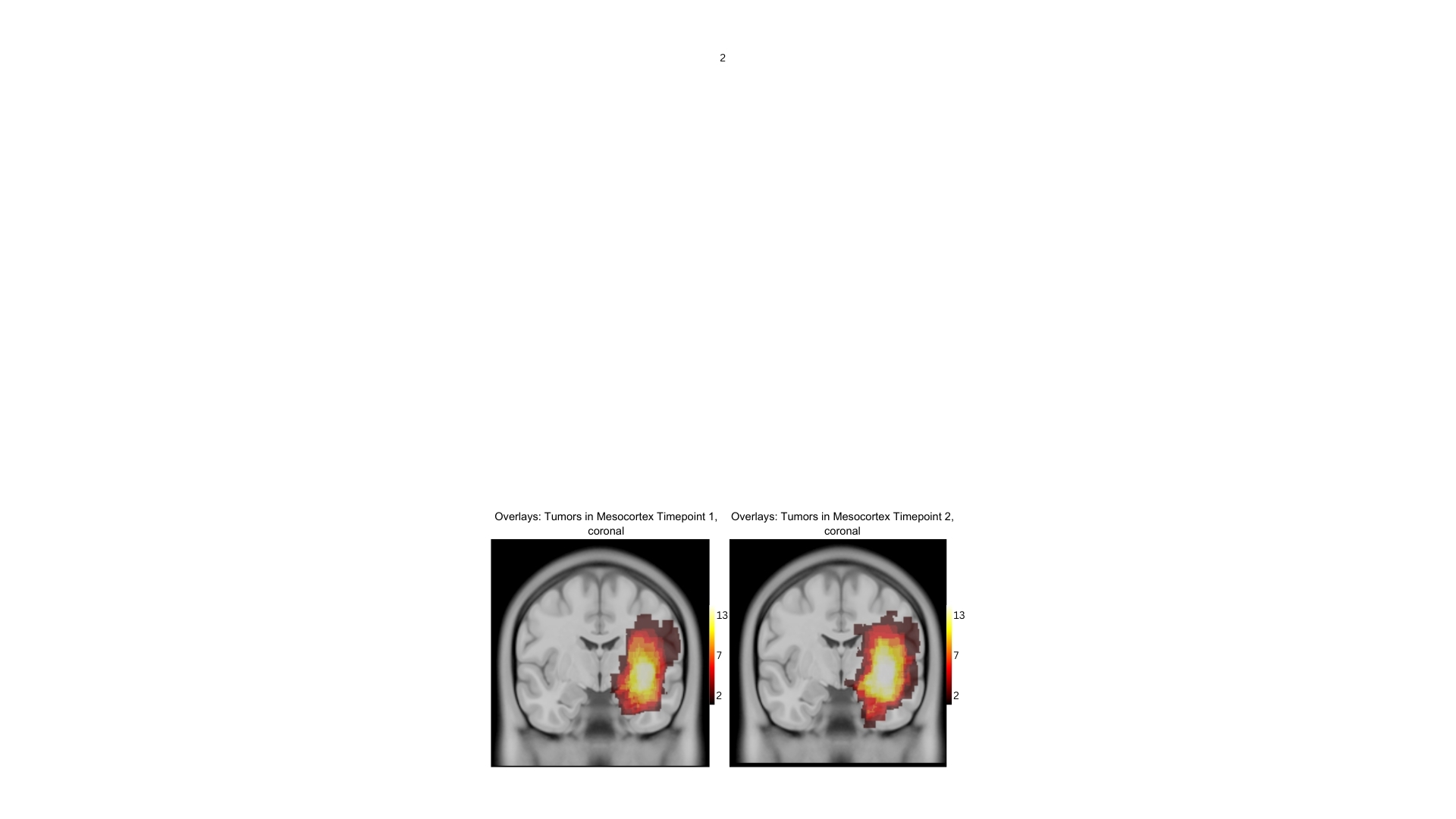
**Figure 3:** **Mesocortical Insula Glioma Confinement with Dissection Reference**

n

n

Mesocortical insula gliomas are shown in transversal, sagittal, and coronal MRI sections, with corresponding dissection images to underscore the tumors' confined growth patterns. These images depict the gliomas' respect for the boundaries of the ventral striatum. The MNI template-based MRI data highlight the tumors' specific expansion within the mesocortex, sparing medial structures and limiting infiltration to the adjacent transitional zones such as the orbitofrontal cortex and the medial temporal pole (BA 38). The heat map scale indicates the number of patients (n) corresponding to each value.


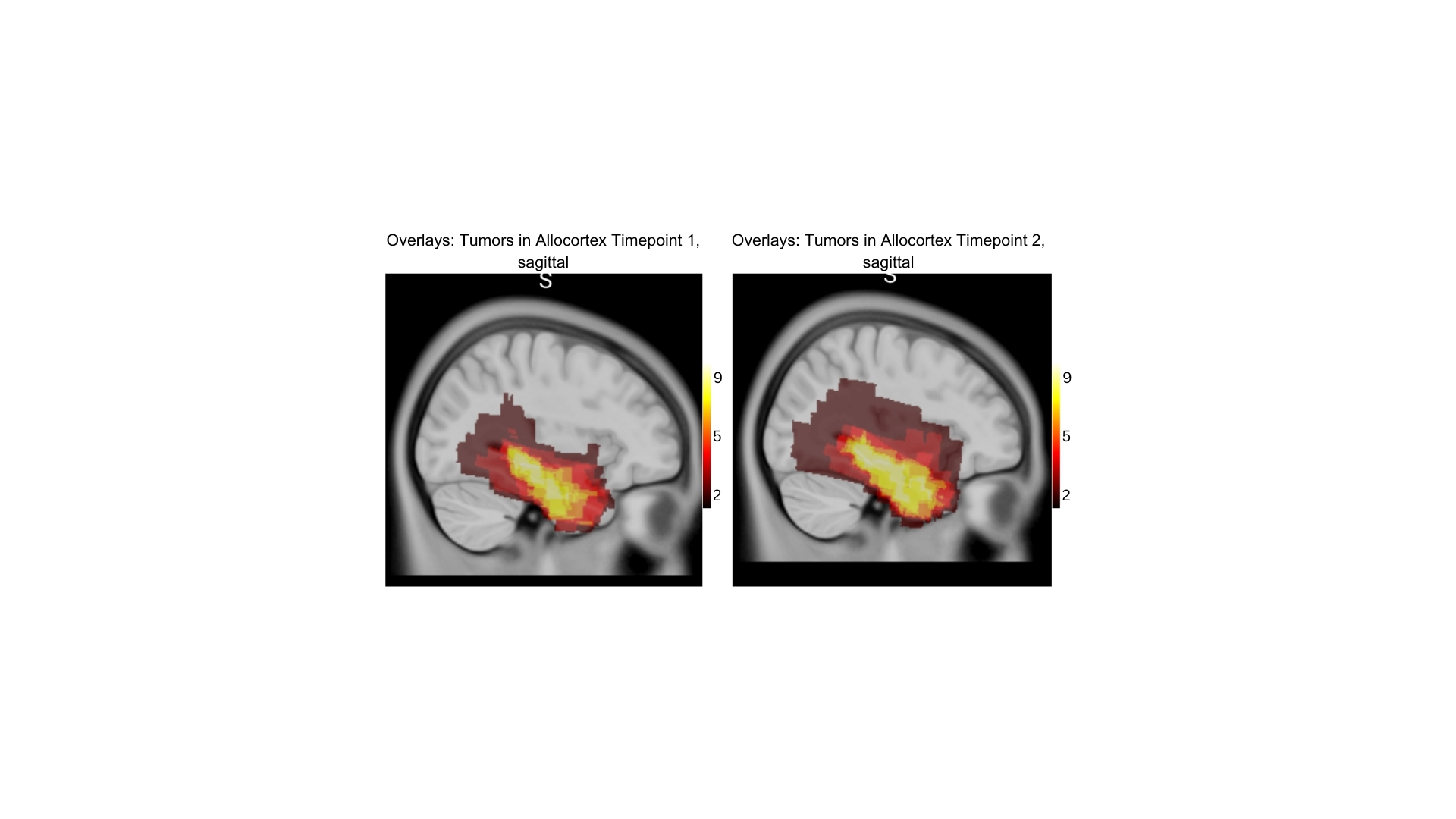

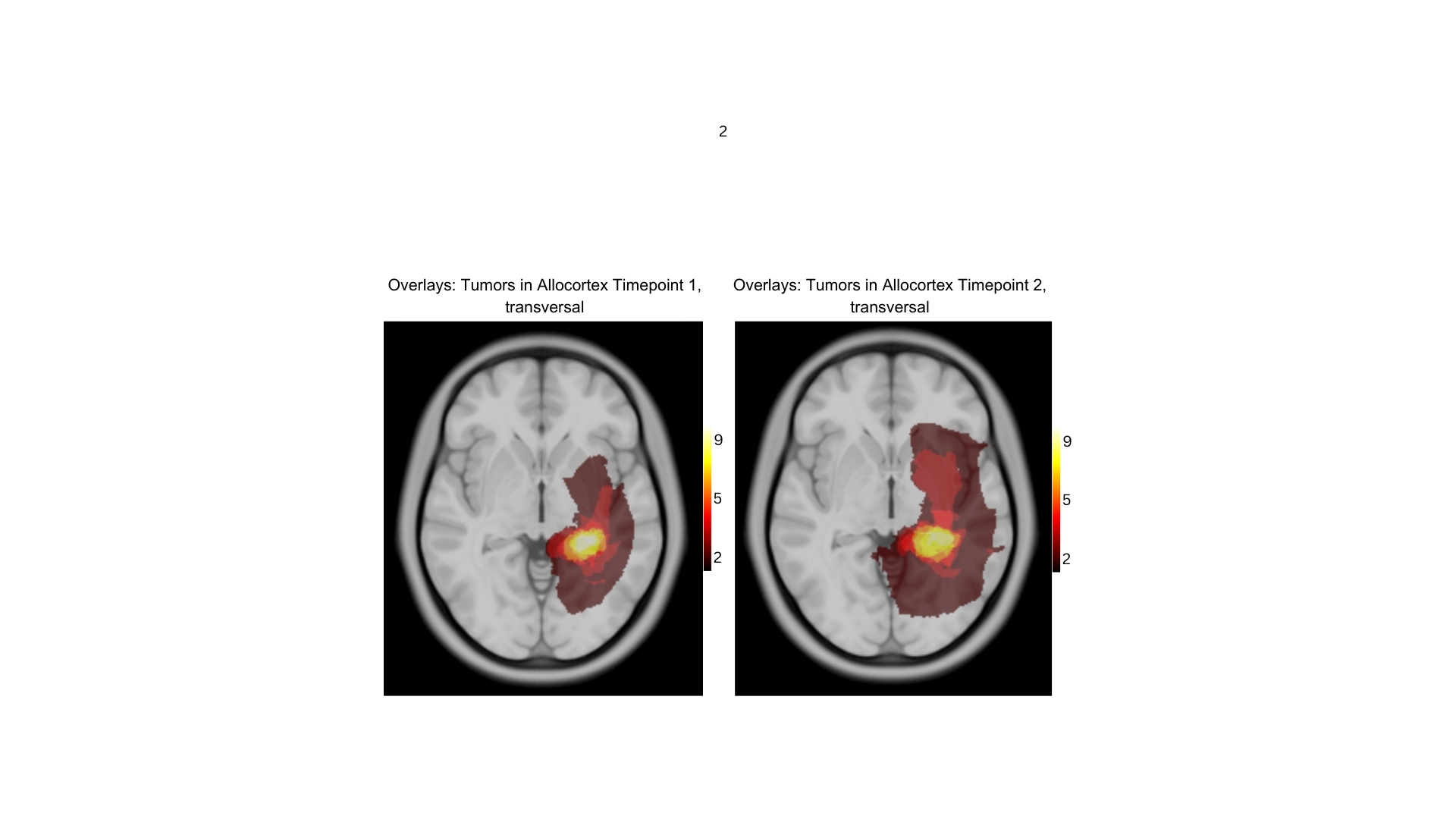


n

n

n

n


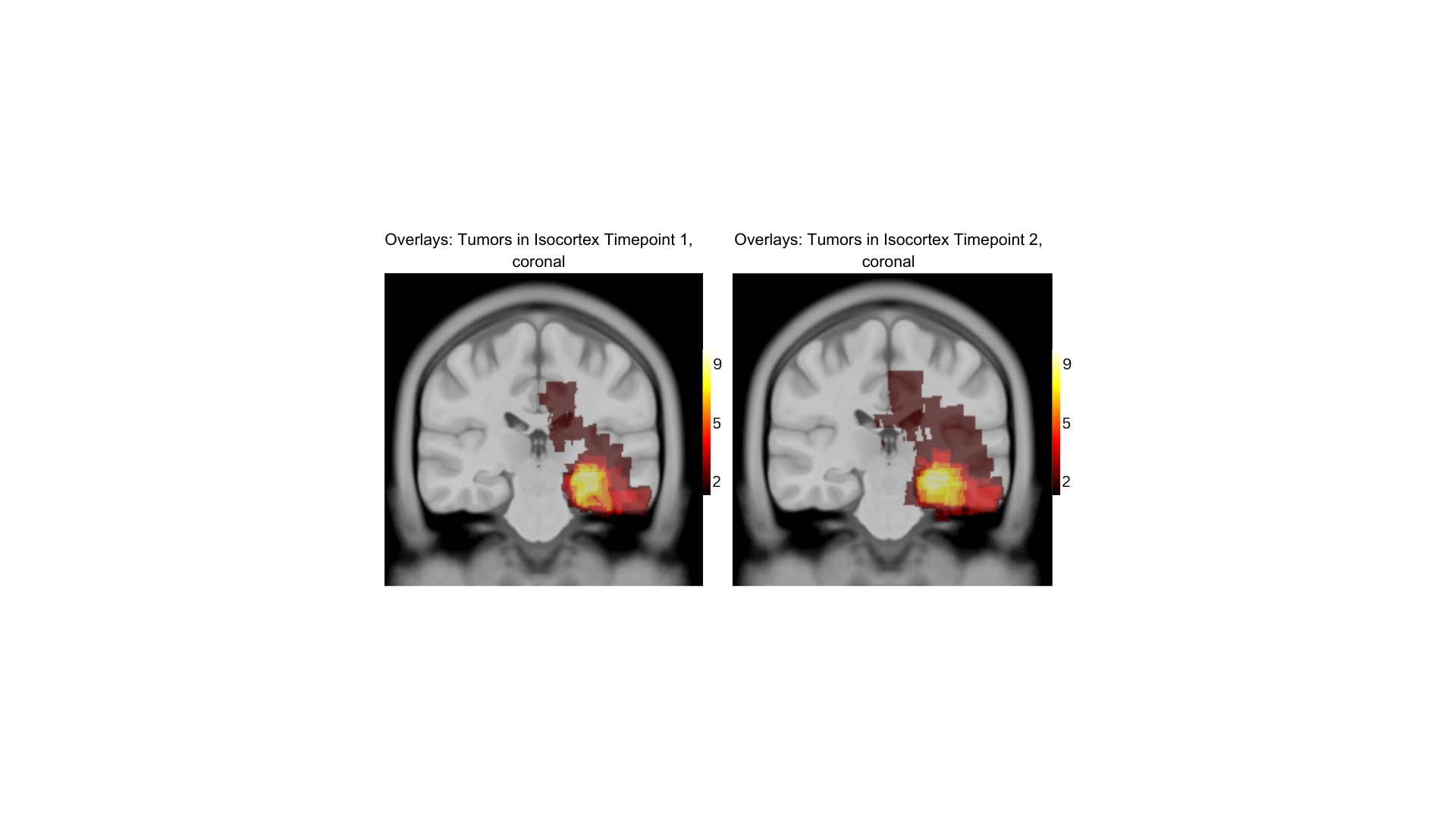


n

n

**Supplementary Figure 4: Allocortical Glioma Progression and Dissection in the Hippocampus** Transversal, sagittal, and coronal MRI scans paired with dissection images illustrate the pathoclistic progression of allocortical gliomas in the hippocampus. The heat maps in T1 and T2 frequencies highlight the gliomas' propensity for longitudinal growth along the hippocampus and encasement of the parahippocampal structures, adhering to the mesiotemporal limbic system. The heat map scale indicates the number of patients (n) corresponding to each value. These images collectively validate the proposed pathoclisis and radioglial expansion concepts herein

**Patient-Specific DTI Analysis**


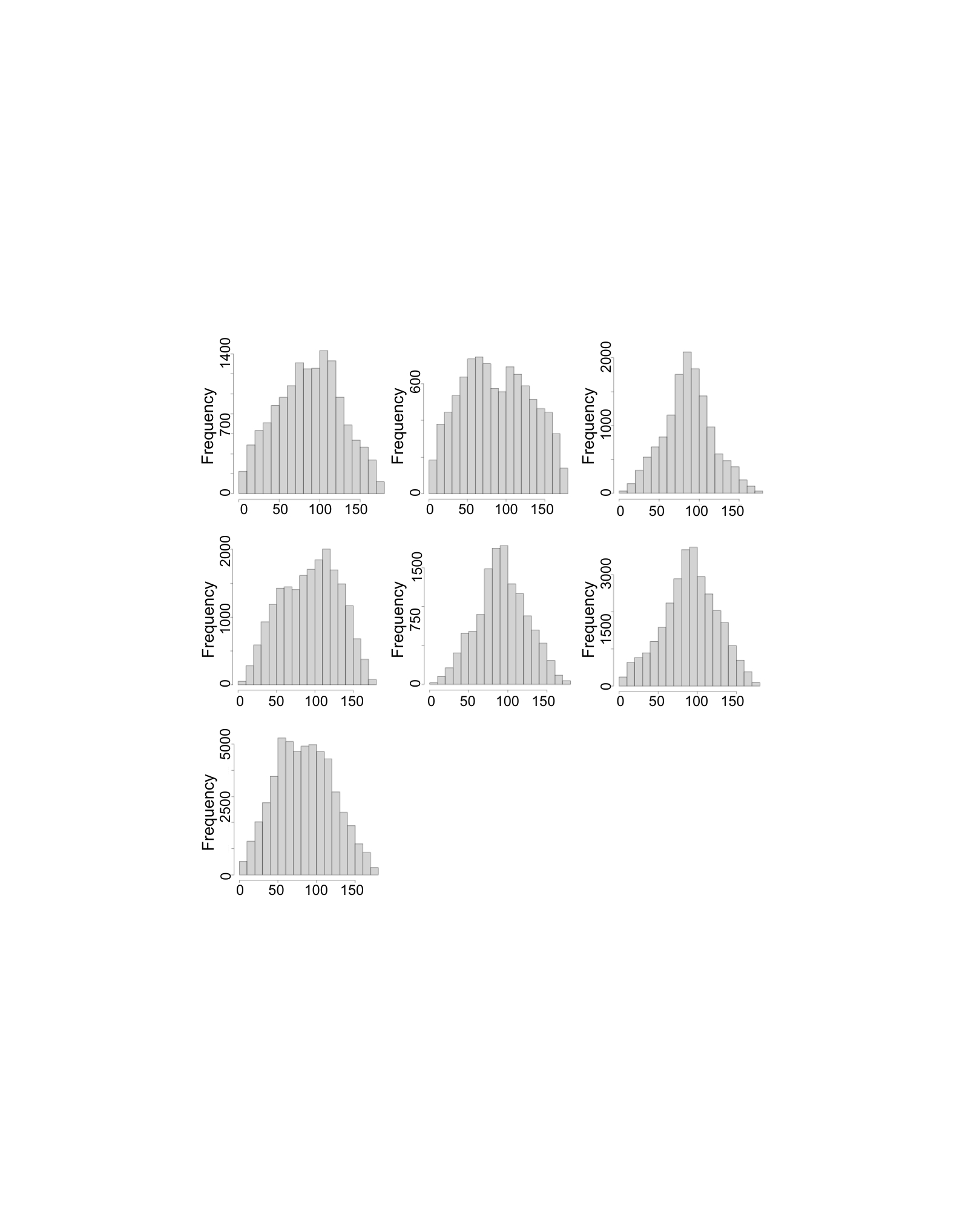


Degrees

Degrees

Degrees

Degrees

Degrees

Degrees

Degrees

**Supplementary Figure 5: Patient-Specific Angular Calculations** Angular calculations were performed voxel-wise using patient-specific DTI data (n=7, 30 diffusion directions, phase-encoding axis [0 1 0], acquisition time of 0.096 seconds, and a b-value of 1000 s/mm²) and their corresponding vector deformation fields. The x‑axis displays angles from 0° to 180°, while the y‑axis shows the frequency of each angle in the analyzed dataset. The results are presented as histograms, showing no angular aggregation within the 0–20-degree range. This indicates a lack of growth tendency along the white matter tracts, consistent with findings from the DTI atlas analysis.

**
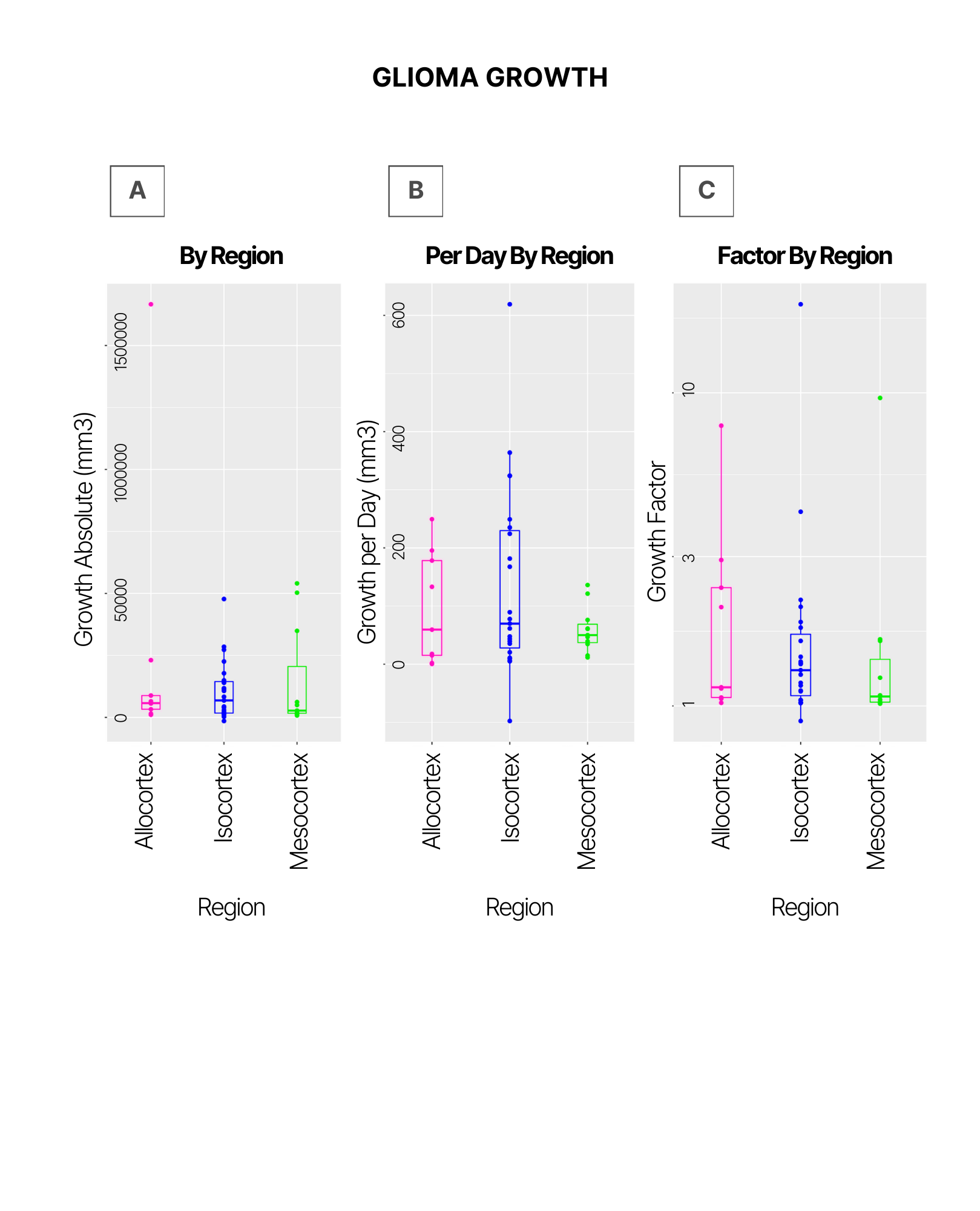
**

**Supplementary Figure 6:** **Box-Plot Analysis of Glioma Growth Metrics in Three Regions.** This figure displays a comparative analysis of glioma growth using three metrics: Absolute Glioma Growth (calculated as Glioma at Time Point 2 minus Glioma at Time Point 1), Daily Glioma Growth Rate (Absolute Growth divided by the number of days between time points), and Glioma Growth Factors (the ratio of glioma size at two time points, also expressible as percentage growth). The data is presented across three distinct regions, using forest plots to effectively highlight growth patterns, regional variations, and confidence intervals for each metric. Each dot represents one patient.

**Tables**

| **Architecture Type** | **Patient Count** | **Male/Female** | **Mean Age Summary (in years)** | **IDH wt** | **IDH mt** | **1p/19q non-codel.** | **1p/19q co-del.** | **WHO II** | **WHO III** |
| --- | --- | --- | --- | --- | --- | --- | --- | --- | --- |
| Allocortex | 9 | 3/6 | 42.89 (21-73) ± 16.46 | 2* | 7 | 7 | 2 | 7 | 2 |
| Isocortex | 24 | 13/11 | 55.42 (27-82) ± 15.08 | 3* | 21 | 10 | 14 | 17 | 7 |
| Mesocortex | 10 | 7/3 | 60.10 (31-86) ± 15.92 | 2* | 8 | 8 | 2 | 8 | 2 |

**Supplementary Table 1:** **Clinicopathological characteristics of the patient cohort.** Seven patients with Astrocytoma IDH wild-type tumors, indicated by *, were included in this study. At the time of histologic evaluation, these tumors exhibited characteristics consistent with a WHO grade II and III glioma according to the WHO 2016 criteria. Based on the newer WHO 2021 classification, these tumors would currently though be categorized as grade IV. No glioblastoma-like radiological features, such as contrast enhancement or necrosis, were observed at diagnosis, thus justifying their inclusion.

**Supplementary Content C**

**Angular Calculations**

Angular alignment between the growth vectors (u) and the principal eigenvectors (v) from the DTI atlas was calculated for each voxel using the formula:

$$\theta=\cos^{-1} \left( \frac{\boldsymbol{u}\cdot\boldsymbol{v}}{|\left| \boldsymbol{u} \right||\cdot|\left| \boldsymbol{v} \right||} \right)$$

The angles, ranging from 0 to 180 degrees, were interpreted such that both 0 and 180 degrees indicated alignment of tumor growth with white matter tracts. To facilitate a more intuitive understanding of alignment, the angles were transformed using the following rule:


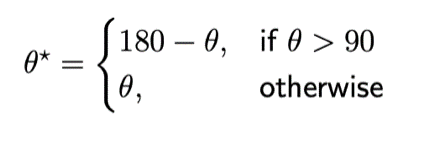


This transformation yielded angles (θ⋆) ranging from 0 to 90 degrees, simplifying the interpretation such that values now indicated how closely tumor growth direction aligned with or were perpendicular to white matter tracts

**Supplementary Figure 7:** Angles between Tumor Growth and White matter tracts: Each plot shows two directional vectors representing the direction of tumor growth (in blue) and the principal orientation of white matter tracts (in red). The bold value is the angle between the two vectors, and the value in regular font indicates the acute angle between the white matter tract and the tumor growth direction. As the orientation of the white matter tract is arbitrary only the acute angle is relevant for the analysis of angular alignment

**Statistical Analysis of Angular Distribution**

Under isotropic growth the acute vector is distributed with pdf

*f*(*θ*) = sin(*θ*) for *θ* ∈ (0*,π/*2)

where *π* corresponds to 180 degrees.

To assess whether growth is aligned with white matter tracts, the acute angle *θ_ij_* between the principal eigenvector *e_j_*_1_ from the DTI and the growth vector *v_ij_* is determined. It is given as

**
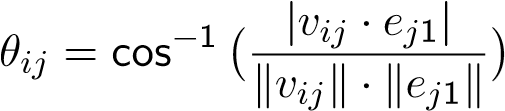
**

Both vectors *v_ij_* and *e_j_*_1_ start at the origin and are scaled to unit length - *e_j_*_1_ is aligned with the zenith reference direction. Thus, the endpoint **x***_ij_* of *v_ij_* lies on the unit sphere, i.e.

∥*xij*∥ = *x*2*ij,*1 + *x*2*ij,*2 + *x*2*ij,*3 = 1*.*

In case of isotropy the distribution of **x***_ij_* is uniform on the surface of the half unit sphere.

The probability that the angle *θ* between glioma growth and the dominant DTI eigenvector is smaller than *θ*_0_ is then given by the corresponding area of the surface of the unit half sphere. This is a spherical cap with polar angle *θ*, if *e_j_*_1_ is aligned with the zenith reference direction.

For a unit sphere the area of a spherical cap with polar angle *θ* is given as 2*π*(1 − cos(*θ*)) and the total area of the half sphere is 2*π*.

Hence under a uniform distribution on the surface of a unit sphere (i.e. no preferred direction of tumor growth with respect to white matter tracts) the cdf of the angle *θ* is given as

*F*(*θ*) = 1 − cos(*θ*)*,*

and hence its pdf is

*f*(*θ*) = sin(*θ*) for *θ* ∈ (0*,π/*2) (*π* corresponds to 180 degrees).

To assess whether growth is aligned with white matter tracts for a first graphical assessment the histogram of the angles *θ_ij_* computed for patient i=1,…,n in voxel j=1, .., m(m=181 x 217 x 181) was overlaid by the pdf *f*(*θ*) under isotropic growth.

To assess whether this alignment is in an angle smaller than 20 degrees (which corresponds to *π/*9) the relative frequency of the proportion of angles smaller than *π/*9,


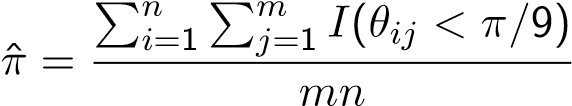


is compared to the probability under isotropy which is given as

*P*(*θ < π/*9) = 1 − cos(*π/*9) = 0,06030738= *p*_0._

Testing the hypothesis that the probability p that the angular alignment of tumor growth and white matter tracts is below 20 degrees corresponds to testing the hypotheses *H*_0_ : p = *p*_0_ versus *H*_1_ : p$\neq$ p_0_  which can be accomplished by a Binomial test . *H*_0_ is rejected at significance level *α*, if *h* is larger than the two-sided critical value. *As N* = *nm* is large the normal approximation can be used to determine the critical value.

The proportion of *θ_ij_* ≤ 20 degrees was computed overall, per subject (including subgroups of subjects) and per voxel.

**Interrater Evaluation**

**
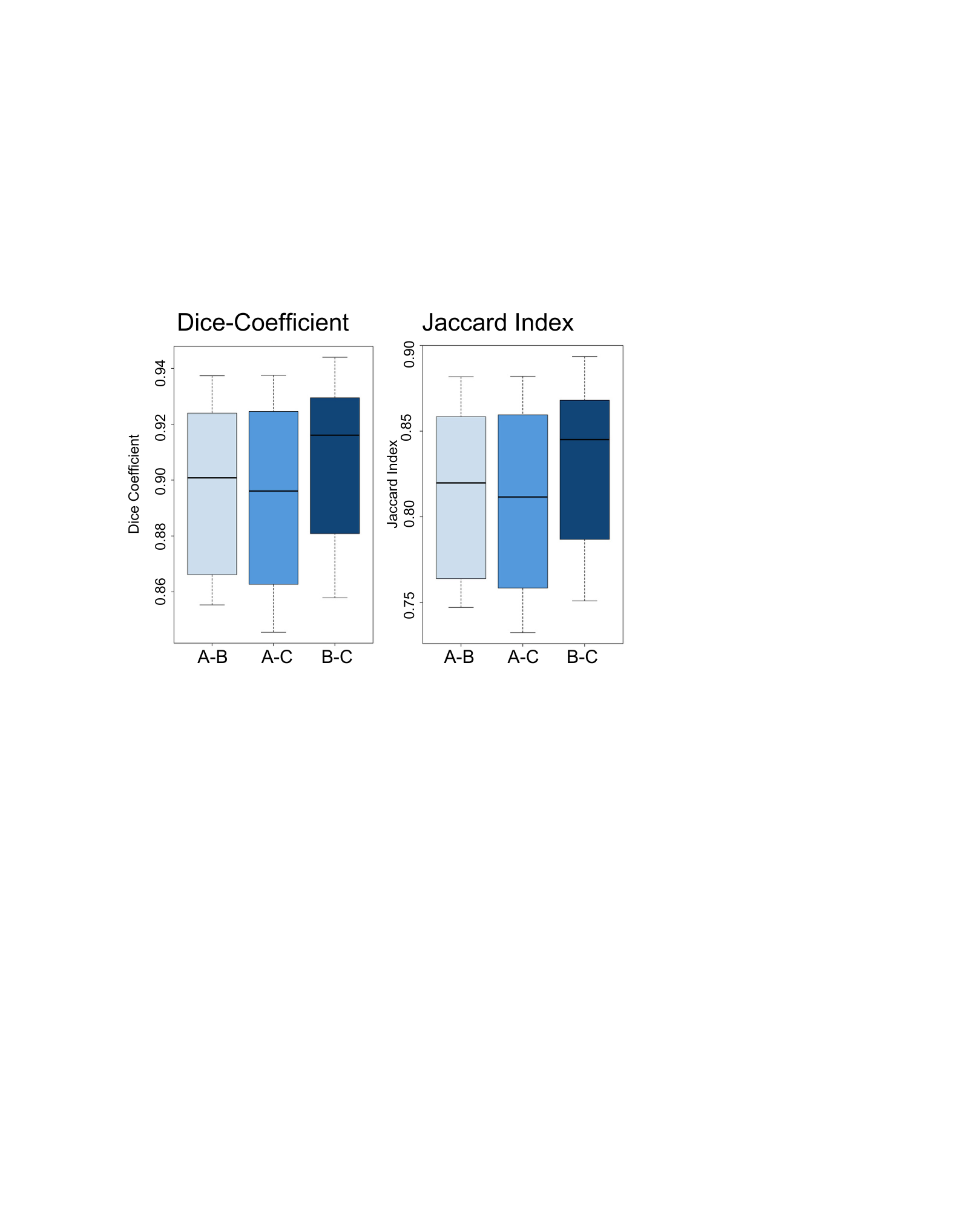
**

**Supplementary Figure 8: Interrater Segmentation Reproducibility.**
Box plots display pairwise segmentation performance assessed by Dice coefficients (left panel) and Jaccard indices (right panel) among three independent raters (A–C). Mean Dice scores were 0.897 (A–B), 0.896 (A–C), and 0.907 (B–C) with standard deviations of 0.031, 0.031, and 0.027, respectively. Mean Jaccard indices were 0.814 (A–B), 0.813 (A–C), and 0.831 (B–C) with standard deviations of 0.051, 0.051, and 0.045, respectively. These high values underscore the excellent reproducibility and consistency of the segmentation method.
